# The long-term impact of the Leprosy Post-Exposure Prophylaxis (LPEP) program on leprosy incidence: A modelling study

**DOI:** 10.1371/journal.pntd.0009279

**Published:** 2021-03-31

**Authors:** David J. Blok, Peter Steinmann, Anuj Tiwari, Tanja Barth-Jaeggi, Mohammad A. Arif, Nand Lal Banstola, Rabindra Baskota, David Blaney, Marc Bonenberger, Teky Budiawan, Arielle Cavaliero, Zaahira Gani, Helena Greter, Eliane Ignotti, Deusdedit V. Kamara, Christa Kasang, Pratap R. Manglani, Liesbeth Mieras, Blasdus F. Njako, Tiara Pakasi, Unnati R. Saha, Paul Saunderson, W. Cairns S. Smith, René Stäheli, Nayani D. Suriyarachchi, Aye Tin Maung, Tin Shwe, Jan van Berkel, Wim H. van Brakel, Bart Vander Plaetse, Marcos Virmond, Millawage S. D. Wijesinghe, Ann Aerts, Jan Hendrik Richardus

**Affiliations:** 1 Department of Public Health, Erasmus MC, University Medical Center Rotterdam, Rotterdam, The Netherlands; 2 Swiss Tropical and Public Health Institute, Basel, Switzerland; 3 University of Basel, Basel, Switzerland; 4 NLR, New Delhi, India; 5 NLR, Kathmandu, Nepal; 6 Ministry of Health and Population of Nepal, Kathmandu, Nepal; 7 Centers for Disease Control and Prevention, Atlanta, Georgia, United States of America; 8 FAIRMED, Bern, Switzerland; 9 NLR, Jakarta, Indonesia; 10 Novartis Foundation, Basel, Switzerland; 11 Universidade do Estado de Mato Grosso, Cáceres, Brasil; 12 National Tuberculosis and Leprosy Program, Dodoma, Tanzania; 13 German Leprosy and Tuberculosis Relief Association, Würzburg, Germany; 14 NLR, Amsterdam, The Netherlands; 15 German Leprosy and Tuberculosis Relief Association, Dar es Salaam, Tanzania; 16 Ministry of Health of the Republic of Indonesia, Jakarta, Indonesia; 17 American Leprosy Missions, Greenville, South Carolina, United States of America; 18 University of Aberdeen, Aberdeen, United Kingdom; 19 FAIRMED, Colombo, Sri Lanka; 20 American Leprosy Missions, Yangon, Myanmar; 21 Instituto Lauro de Souza Lima & UNINOVE, Bauru, Brazil; 22 Anti-Leprosy Campaign, Colombo, Sri Lanka; Colorado State University - Global Campus, UNITED STATES

## Abstract

**Background:**

The Leprosy Post-Exposure Prophylaxis (LPEP) program explored the feasibility and impact of contact tracing and the provision of single dose rifampicin (SDR) to eligible contacts of newly diagnosed leprosy patients in Brazil, India, Indonesia, Myanmar, Nepal, Sri Lanka and Tanzania. As the impact of the programme is difficult to establish in the short term, we apply mathematical modelling to predict its long-term impact on the leprosy incidence.

**Methodology:**

The individual-based model SIMCOLEP was calibrated and validated to the historic leprosy incidence data in the study areas. For each area, we assessed two scenarios: 1) continuation of existing routine activities as in 2014; and 2) routine activities combined with LPEP starting in 2015. The number of contacts per index patient screened varied from 1 to 36 between areas. Projections were made until 2040.

**Principal findings:**

In all areas, the LPEP program increased the number of detected cases in the first year(s) of the programme as compared to the routine programme, followed by a faster reduction afterwards with increasing benefit over time. LPEP could accelerate the reduction of the leprosy incidence by up to six years as compared to the routine programme. The impact of LPEP varied by area due to differences in the number of contacts per index patient included and differences in leprosy epidemiology and routine control programme.

**Conclusions:**

The LPEP program contributes significantly to the reduction of the leprosy incidence and could potentially accelerate the interruption of transmission. It would be advisable to include contact tracing/screening and SDR in routine leprosy programmes.

## Author summary

The Leprosy Post-Exposure Prophylaxis (LPEP) program explored the feasibility and impact of contact tracing and the provision of SDR to eligible contacts of newly diagnosed leprosy patients in states or districts of Brazil, India, Indonesia, Myanmar, Nepal, Sri Lanka and Tanzania. This study investigated the long-term impact of the LPEP program on the leprosy new case detection rate (NCDR). Our results show that LPEP could reduce the NCDR beyond the impact of the routine leprosy control programme and that many new cases could be prevented. The benefit of LPEP increases gradually over time. LPEP could accelerate the time of reaching predicted NCDR levels of 2040 under routine program by up to six years. Furthermore, we highlighted how the impact varies between countries due to differences in the number of contacts per index patient screened and differences in leprosy epidemiology and national control programme. Generally, including both household contacts and neighbours (> 20 contacts per index patient) would yield the highest impact.

## Introduction

Leprosy is a chronic infectious disease caused by *Mycobacterium leprae*. The mode of transmission is most likely through the respiratory tract.[1,2] Close contacts of leprosy patients have the highest risk of infection with *M*. *leprae* and developing the disease.[3] However, only a small proportion of an infected population develops the disease. Leprosy is characterized by a long incubation period, sometimes more than a decade.[4] Currently, the detection and diagnosis of leprosy are based on clinical signs and symptoms along with a slit-skin smear. Based on the number of skin lesions leprosy is classified into paucibacillary (PB) and multibacillary (MB) leprosy. MB leprosy is known to be chronic and highly infectious. Early detection and diagnosis of patients, and treatment with multidrug therapy (MDT) have been the mainstay of the current control strategy until recently.[5]

Globally just over 200,000 new cases of leprosy are detected annually.[5] This number has stagnated since leprosy was declared eliminated as a public health problem globally in 2001. It became clear that the current strategy was not sufficient to further decrease the number of new leprosy cases, let alone to interrupt transmission of *M*. *leprae*. New tools and approaches are therefore essential.[6] Currently, contact tracing and screening, and the provision of post-exposure prophylaxis (PEP), usually a single dose of rifampicin (SDR), to contacts of a patient is the most promising option to reduce the risk of developing leprosy among individuals exposed to *M*. *leprae*.[7–9]

The Leprosy Post-Exposure Prophylaxis (LPEP) program was established in 2014.[10,11] The goal was to assess the feasibility and impact of SDR administered to eligible contacts of newly diagnosed leprosy patients. In this three-year programme, household contacts and neighbours of leprosy patients were systematically traced, screened for signs and symptoms of leprosy and given SDR if they did not have leprosy or other contraindications (e.g. signs and symptoms of tuberculosis). Newly detected cases among contacts were enrolled for MDT following the standard leprosy control programme procedures. The LPEP program was implemented in seven countries: Brazil, India, Indonesia, Myanmar, Nepal, Sri Lanka and Tanzania. In most countries, implementation started in 2015 and ended in 2018.

The impact of SDR on the new case detection rate (NCDR) is difficult to establish in a three-year programme. This is due to the existing backlog of undiagnosed leprosy patients and the slow progression of the disease (long incubation time).[12] The transmission dynamics of leprosy are known to be non-linear, i.e. current leprosy incidence is determined by past individual exposure to *M*. *leprae*. Mathematical modelling is a powerful and efficient tool for capturing no-linear transmission dynamics and evaluating long-term effects of interventions.[13] In this study, we use the established individual-based model SIMCOLEP to predict the long-term impact of the LPEP program.[14,15] This model has been used in the past to predict the trend of leprosy incidence in Brazil, India and Indonesia, the impact of SDR in northwest Bangladesh, Pará State in Brazil and Kiribati, and the impact of diagnostics.[16–21] The aim of this study is to estimate the long-term impact of the LPEP program on the NCDR in Brazil, India, Indonesia, Myanmar, Nepal, Sri Lanka and Tanzania.

## Methods

### Model description

We used the individual-based model SIMCOLEP that simulates the spread of *M*. *leprae* in a population structured in households. It models life-histories of individuals, which are born and live in households. Over time, individuals can create their own household or move to another household after marriage, during adolescence or after becoming widowed. Death estimates are based on death rates at birth (Table A, B, and Fig A-F in S1 Text).[14]

In the model, susceptibility of an individual to develop leprosy was randomly allocated, because previous modelling studies showed that a random allocation would yield similar results as other susceptibility mechanisms, such as genetic inheritance.[14,16] We assume that 20% of the population is susceptible, implying that 80% will not develop leprosy. An infectious individual can transmit *M*. *leprae* through contact with a susceptible individual. Two transmission processes are modelled separately: transmission in the general population and within-household. The latter captures the increased risk of household members to acquire the infection. Transmission is determined by the product of the contact rate, both in the general population and within households, and the probability of infection during contact between individuals. The contact rate reflects the rate at which an infectious individual has contact with another (susceptible) individual per year. An infected individual develops either PB or MB leprosy, the frequency of which is assigned based on the proportion of MB leprosy in the country. We assumed that the infectiousness of MB leprosy patients is increasing in the subclinical stage and at the maximum in the clinical stage.

The natural history of leprosy is modelled following Meima et al.[22] After infection, an individual starts with an asymptomatic incubation period, which on average lasts 4.2 years for PB and 11.1 years for MB (standard deviation 1.9 and 5.0 years, respectively).[4,22] Afterwards the individual proceeds to a symptomatic infection. A symptomatic PB leprosy case can self-heal with a rate of 20% per year.[23] An MB leprosy case remains symptomatic and infectious until treatment or death (Table C in S1 Text).

Leprosy control encompasses detection of patients through passive case detection and may include active case finding activities, and treatment of diagnosed leprosy patients with MDT. Passive case detection is reflected in the model by annual detection delays, which are estimated during the calibration process. Active case finding activities include contact tracing and screening, and population surveys (i.e. door-to-door). In the model, these activities are defined by year, and assigned a certain coverage rate. After detection, a leprosy patient is enrolled for MDT treatment and assumed to be no longer infectious after treatment. In the model, relapses occur with a rate of 0·001 per year: 90% relapses to MB and 10% to PB (Table E in S1 Text).[24,25] We further included a protective effect of 60% for those contacts with a previous BCG vaccination (Fig H in S1 Text).[26] A full description of the model can be found in S1 Model description, and Fischer et al. and Blok et al.[14,15]

### Model fitting and validation

The model was fitted to the leprosy situation in states or districts of seven LPEP countries: 1) Alta Floresta city and region and Rondonópolis city in Mato Grosso, Araguaína and Colinas do Tocantins in Tocantins, and Petrolina city and region in Pernambuco, Brazil; 2) Union Territory Dadra and Nagar Haveli, India; 3) Sumenep district, Indonesia; 4) Nyaung Oo, Myingyan and Tharyarwaddy Townships, Myanmar; 5) Jhapa, Morang and Parsa districts, Nepal; 6) Kalutara and Puttalam districts, Sri Lanka; and 7) Kilombero, Liwale and Nanyumbu districts, Tanzania. These subnational areas are henceforth addressed as Brazil, India, Indonesia, Myanmar Nepal, Sri Lanka and Tanzania.

First, we quantified the population of each area using area- or country-specific demographic data as input, including population growth rates, birth rates, death rates, fertility rates and the age distribution. These data were obtained from various sources including country census, Demographic and Health surveys (DHS) and the World Health Organization (WHO). Second, the household size distribution was fitted to the observed distribution. Parameters that determine formation and changes of households were calibrated for each LPEP area (Table B and Fig F in S1 Text).

After considering the household size distribution, the model was fitted to replicate the leprosy new case detection rate (NCDR) trends in each LPEP area. Leprosy epidemiologic data were obtained from national leprosy reports (Section B in S1 Text). Area specific historical data on leprosy NCDR and the MB proportion were available for all LPEP program areas, except for Myanmar where instead, country level data was used (Fig G in S1 Text). The area specific MB proportion was: Brazil (69%), India (26%), Indonesia (76%), Myanmar (77%), Nepal (48%), Sri Lanka (47%) and Tanzania (71%).

The NCDR was fitted to the data by calibrating the contact rate and detection delays. The contact rate determines largely the level of endemicity of the modelled setting. We only calibrated the contact rate in the general population. The contact rate within households was fixed to 0.98 based on a previous publication.[14] We assumed that although household sizes differ between countries and regions, the contact rate within households would not (Table D in S1 Text).

In the model, detection delays reflect the intensity of the existing activities of the leprosy control programme in place. Any improvement in leprosy case detection is reflected by a decrease in the detection delay. Based on the historical NCDR trend, we identified for each LPEP area the years in which the NCDR increased, assuming this was the result of improved (passive) case finding. Changes in detection delays in the identified years were assumed to follow a logistic function, which was calibrated to match the observed NCDR trend. Additionally, if an area had implemented active case finding activities in a certain year, this was also included in the model. In that case, the coverage of active case finding was calibrated to match the increase in the observed NCDR (Table E in S1 Text).

For the calibration, we randomly drew parameter values from uniform distributions with intervals wide enough to capture all possible values that could produce a good fit (Table F in S1 Text). The model was run with these parameter values. The goodness of fit of a run was assessed using a log-likelihood assuming a Poisson distribution. A parameter combination was accepted when the log-likelihood did not deviate from the maximum log-likelihood more than 1.5 times. We repeated this until 1,000 parameter combinations were accepted. Uncertainty intervals, which reflect uncertainty in the parameter values, were calculated by discarding the 2.5% highest and lowest values.

To validate the model, we first calibrated it using NCDR data until 2012 only (Fig I in S1 Text). We then evaluated the model’s the ability to forecast the data points in 2013 and 2014 (Fig J in S1 Text). All data points lie within the distribution of short-term forecasted NCDR, indicating a good forecast. We excluded the data of 2015 and beyond because in these years the LPEP program had been implemented. After model validation, we fitted the model to the data until 2014 (Fig K and L in S1 Text), and then validated predictions of LPEP with the data beyond 2015 (Fig M in S1 Text). The fitted model was set to reflect the routine programme before the LPEP program was introduced.

### Scenarios

Two scenarios were modelled: 1) the continuation of the routine programme as in 2014 (i.e. counterfactual), and 2) the addition of the LPEP program activities. For the routine programme, we forecasted the NCDR of the fitted model until 2040 for each LPEP area. The routine programme from 2014 onwards relied on passive case detection only, except for India where an active case finding campaign was started in 2015.

The LPEP program started in 2015, except in Sri Lanka and Brazil where it started in 2016. Household contacts and neighbours of diagnosed patients were traced and screened. Only eligible contacts without clinical leprosy were given SDR.[10] Based on results from the COLEP trial, we assumed that the effectiveness of SDR was higher among neighbours and social contacts (70%) than household contacts (50%).[8] The LPEP program was further characterized by retrospective contact tracing, i.e. tracing and screening of contacts of patients diagnosed prior to the start of the program.[10] The actual implementation of the LPEP program varied between the LPEP areas with respect to the starting year, retrospective contact tracing period and the number of contacts per index patient that were included (Table 1 and Table F in S1 Text). Predictions of the NCDR under the LPEP program were made until 2040 for each LPEP area.

**Table 1:** Area-specific LPEP program characteristics as quantified in the model.

LPEP area	Starting year	Retrospective period in years	Contacts per index patient (mean)	
			Listed	Screened
Brazil	2016	1	12	11
India	2015	2	26	26
Indonesia	2015	0.5	37	36
Myanmar	2015	1	19	18
Nepal	2015	2	23	23
Sri Lanka	2016^a^	1	2^b^	1^b^
Tanzania	2015	1	10^b^	9^b^

a Actual start date Dec 2015

b Only household contact(s) were accepted for screening because of the high level of social stigma associated with leprosy

## Results

**Fig 1:**
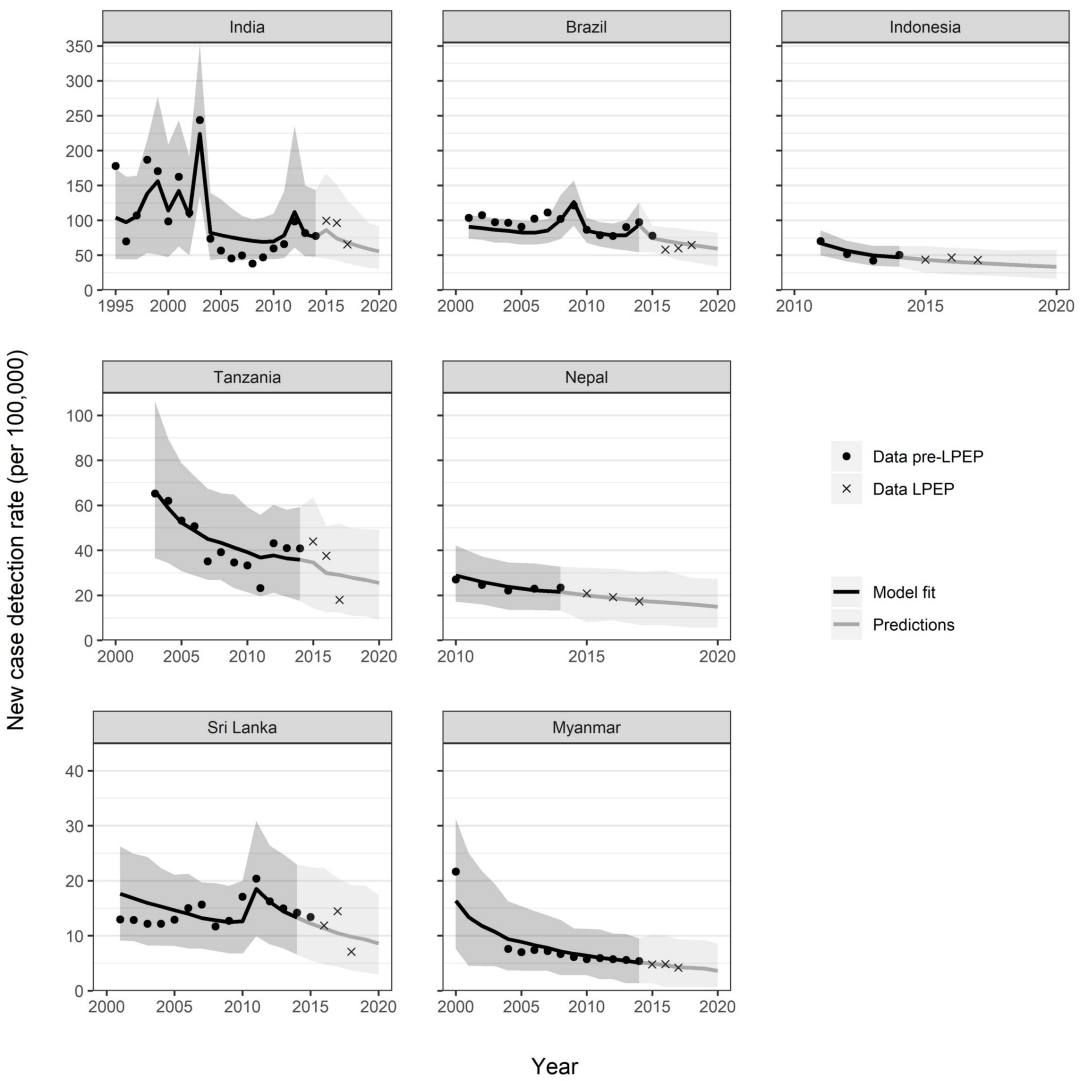
Comparison of modelled trends with the observed leprosy new case detection rates in LPEP areas across states and districts in seven countries. Each panel represents the trends in subnational states or districts (arranged in descending order of baseline endemicity level): India (Union Territory Dadra and Nagar Haveli); Brazil (Alta Floresta city and region and Rondonópolis city in Mato Grosso, Araguaína and Colinas do Tocantins in Tocantins, Petrolina city and region in Pernambuco); Indonesia (Sumenep district); Tanzania (Kilombero, Liwale and Nanyumbu); Nepal (Jhapa, Morang and Parsa districts); Sri Lanka (Kalutara and Puttalam districts); Myanmar (Nyaung Oo, Myingyan and Tharyarwaddy Townships). Data are represented by black dots and crosses. The solid line represents the mean model estimate and the shaded area the 95% prediction interval.

Fig 1 shows the mean and 95% uncertainty interval of the predicted NCDR for all LPEP areas as compared to the observed data points. The model provides a good visual fit to the observed NCDR. The level of endemicity varied between the LPEP areas. In 2015 the modelled NCDR per 100,000 was 86 in India, 75 in Brazil, 43 in Indonesia, 35 in Tanzania, 20 in Nepal, 12 in Sri Lanka, and 5 in Myanmar.

**Fig 2:**
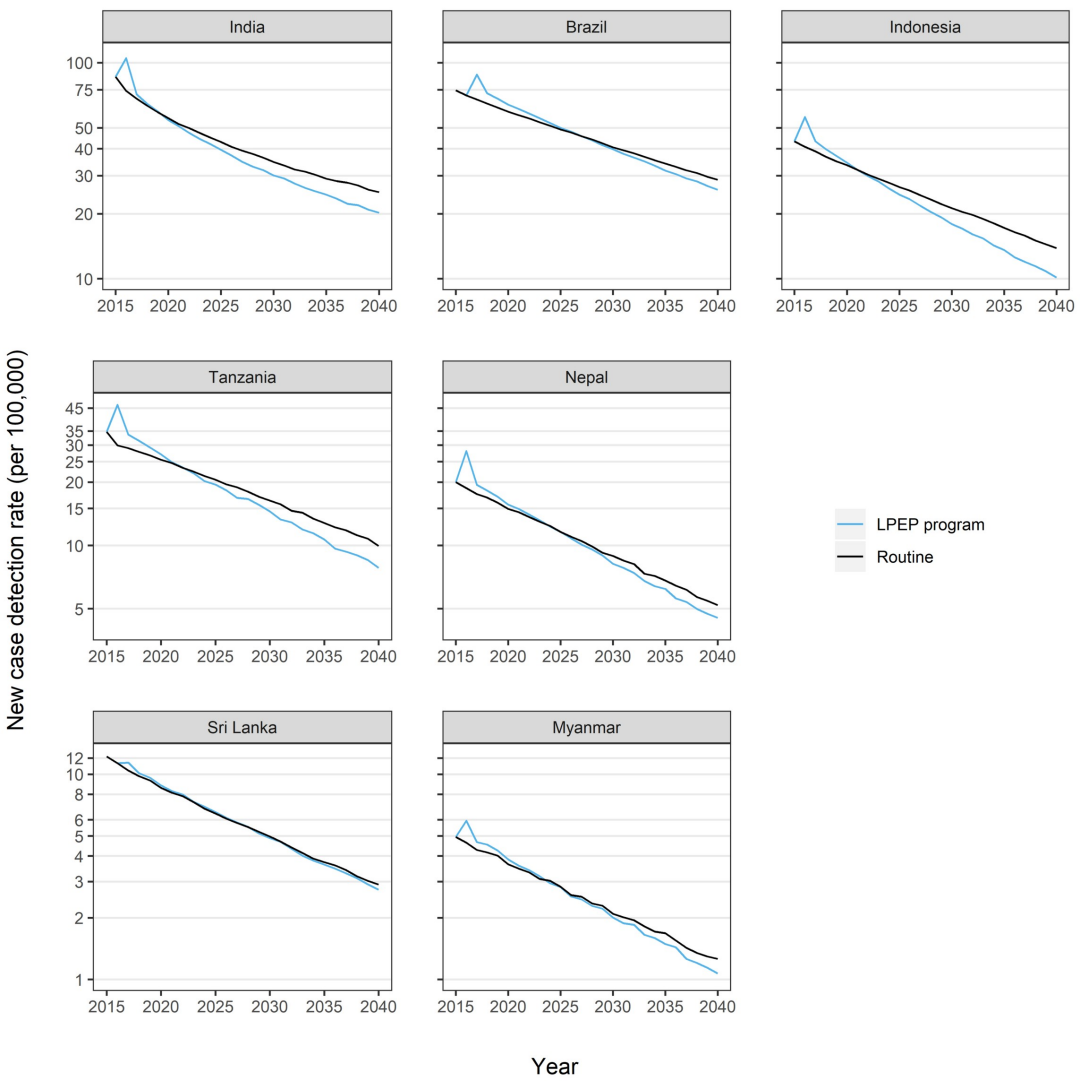
Predicted long term trends of the leprosy new case detection rate under the routine programme, and combined with the LPEP program, stratified by LPEP area. Each panel represents the trends in subnational states or districts (arranged in descending order of baseline endemicity level): India (Union Territory Dadra and Nagar Haveli); Brazil (Alta Floresta city and region and Rondonópolis city in Mato Grosso, Araguaína and Colinas do Tocantins in Tocantins, Petrolina city and region in Pernambuco); Indonesia (Sumenep district); Tanzania (Kilombero, Liwale and Nanyumbu); Nepal (Jhapa, Morang and Parsa districts); Sri Lanka (Kalutara and Puttalam districts); Myanmar (Nyaung Oo, Myingyan and Tharyarwaddy Townships). Model predictions are represented by means of 1,000 repeats (solid line). The blue line represents the LPEP program and the black line the routine programme.

Fig 2 shows the long-term impact of the LPEP program versus the routine scenario by study area. Generally, the LPEP program prediction is characterized by an initial increase in the NCDR, because of the active case finding activities. The extent of this increase varies between study areas due to differences in the retrospective period and the number of contacts screened per index patient. Later, the NCDR declines at a higher rate as compared to the routine scenario. The modelled NCDR in the LPEP program drops below the routine scenario after four to ten years, depending on the study area. In Sri Lanka, only minimal benefits attributable to LPEP could be observed. If the LPEP program were continuously implemented at comparable intensity, we predicted a drop in the NCDR of 55% (vs. 40% under routine) in Brazil, 71% (vs. 53%) in India, 68% (vs. 48%) in Indonesia, 66% (vs. 55%) in Myanmar, 71% (vs. 52%) in Nepal, 57% (vs. 52%) in Sri Lanka, and 69% (vs. 45%) in Tanzania by 2030. Over time, the model showed an increasing beneficial effect of the LPEP program as compared to the routine control programme in all study areas.

As a direct public health benefit, the LPEP program activities have the potential to reduce the time of reaching the modelled NCDR levels of 2040 of our routine scenario. In India and Indonesia, the NCDR level of 2040 under the routine scenario could be achieved already in 2034 with LPEP; thereby accelerating progress by six years. In the remaining study areas, the time of achieving the NCDR level of 2040 could be reduced by five years in Tanzania, three years in Brazil, Nepal and Myanmar, and one year in Sri Lanka.

**Fig 3:**
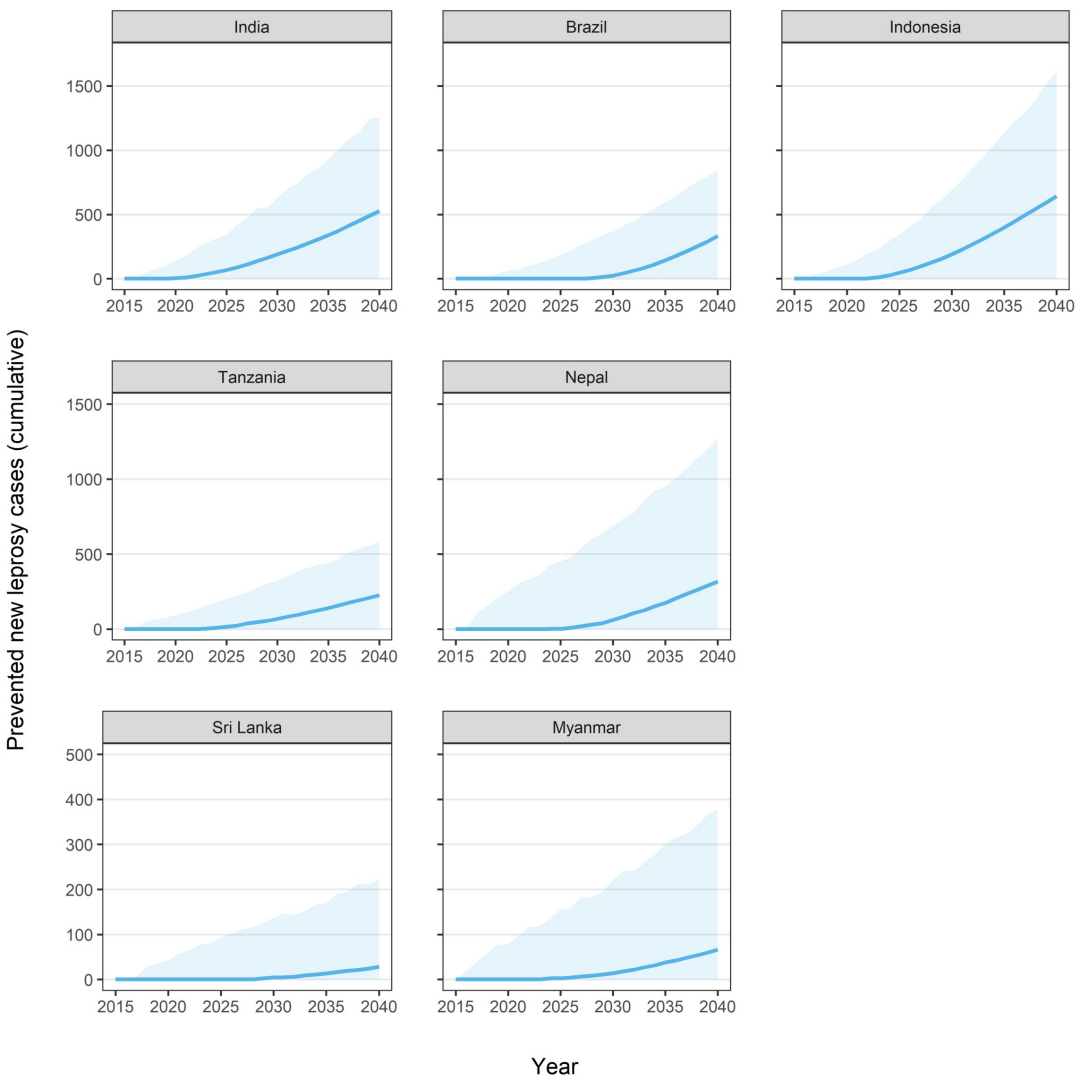
Number of cumulative new leprosy cases prevented due to the LPEP program activities as compared to the routine programme alone. Each panel represents the trends in subnational states or districts (arranged in descending order of baseline endemicity level): India (Union Territory Dadra and Nagar Haveli); Brazil (Alta Floresta city and region and Rondonópolis city in Mato Grosso, Araguaína and Colinas do Tocantins in Tocantins, Petrolina city and region in Pernambuco); Indonesia (Sumenep district); Tanzania (Kilombero, Liwale and Nanyumbu); Nepal (Jhapa, Morang and Parsa districts); Sri Lanka (Kalutara and Puttalam districts); Myanmar (Nyaung Oo, Myingyan and Tharyarwaddy Townships). Model predictions are represented by means of 1,000 repeats (solid line). The blue line represents the LPEP program. The shaded area is the 95% prediction interval.

Fig 3 shows the number of new leprosy patients that could be prevented through the LPEP program activities as compared to the routine programme. In the first years no cases were prevented as a result of the increased NCDR due to the existing backlog of undiagnosed patients (Fig 2) and the long incubation period. Afterwards, the number of prevented cases increased exponentially in all study areas. According to our predictions, the mean number of new cases prevented in the long run varied by study area: 330 (0–840) in Brazil, 530 (0–1260) in India, 640 (0–1610) in Indonesia, 70 (0–380) in Myanmar, 320 (0–1270) in Nepal, 30 (0–220) in Sri Lanka, and 230 (0–580) in Tanzania. In total, 2,150 (0–6,160) new cases in a population of around 10 million (i.e., 21.5 per 100,000) could be prevented over a period of 25 years.

## Discussion

Our modelling study shows that the LPEP program activities, which include contact tracing and screening as well as the provision of SDR to eligible contacts, in the long-term, potentially reduce the number of new leprosy cases beyond the impact of the routine leprosy control programme. Although SDR-PEP has an immediate impact on preventing subclinical cases to develop clinical leprosy, this cannot be observed in the NCDR directly due to the long incubation period. Generally, the NCDR increases in the first years due to the detection of previously undiagnosed cases in the frame of retro- and prospective contact tracing and screening (active case finding). After a peak, the NCDR starts to decrease at a higher rate as compared to the routine programme. The benefit of LPEP thus increases gradually over time. The LPEP program activities could accelerate achieving certain set (lower) NCDR goals, and prevent a substantial number of new cases.

Across the LPEP study areas, we observe marked differences in terms of impact. These differences depend firstly on the intensity of the LPEP program implementation. As expected, the number of contacts screened per index patient determines to a large extent the rate of decline in NCDR. For example, India and Indonesia included the highest number of contacts and consequently achieved a substantial reduction in the NCDR, while Sri Lanka recorded a minimal impact on NCDR as a result of including on average only one contact per patient due to high social stigma and logistical constraints. Secondly, differences in impact can also be explained by the level of endemicity and the quality of routine leprosy programme. In areas with a higher level of endemicity, such as India, more contacts are screened and hence are eligible for SDR, which as a result will prevent more cases in absolute terms as compared to an area with a lower level of endemicity, such as Myanmar (at country level). Furthermore, the impact is larger if the routine leprosy programme is better (reflected by shorter passive case detection delays), which implies that there are less undiagnosed clinical leprosy patients in the population. Undiagnosed clinical leprosy patients continue to contribute to the transmission, making an intervention like LPEP less efficient (Table H in S1 Text).

This study highlights the importance of active case finding to detect undiagnosed clinical leprosy patients in the population. The predicted increase in the number of newly diagnosed leprosy patients in the first years indicates that there is a pool of undiagnosed and hidden patients in the population, which would remain undiagnosed if the LPEP program were not rolled out. This is in line with earlier studies suggesting that many leprosy cases remain hidden or undetected.[12,27] The patients that are diagnosed through contact screening in the frame of the LPEP program would otherwise very likely be detected only years later through passive case detection as modelled for the routine programme. If retro- and prospective contact tracing would have been implemented without SDR-PEP this would result in a higher NCDR during the entire simulation period compared to the routine programme (Fig N in S1 Text). As a result, the lower NCDR of the routine programme is an incomplete reflection of the true burden, whereas the NCDR in the LPEP arm likely gives a more realistic picture of the true endemicity level. Accounting for the missed cases in the routine scenario, the LPEP program would show an even larger impact.

Our model results show that a continuation of LPEP beyond the three-year programme is recommended to achieve a sustained reduction in the NCDR. Our predictions indicate that with time the benefit of the LPEP program increases. At the same time, the investment in terms of contacts needing SDR declines over time, suggesting a possible decline in necessary resources (Fig O in S1 Text). The total number of contacts who receive SDR is highest in the first year, because of relatively high patient numbers further boosted by the retrospective contact tracing. However, as the number of newly diagnosed patients declines as a result of the LPEP program, also less contacts will be needing screening and SDR in the future. In the long run, our model predicted that about a half million contacts (cumulative) would need to receive SDR to prevent 2,150 new cases. A cost-effectiveness study of the LPEP program in India showed that the program was cost-effective in the short (5 years) and long term (25 years).[28]

To optimize the benefit of the LPEP program, it is advisable to trace and screen many contacts per index patient. Areas with a larger number of contacts per patient screened and provided with SDR showed a higher reduction in the NCDR. Moreover, the largest decline in NCDR was observed immediately after the initial peak of NCDR, because at that time point the reach of the intervention programme is the largest (i.e., most contacts are included). Over time the reach of the intervention will drop because of declining numbers, as the program partly rely on passive case detection for the index patient. This could slow down the effects of contact screening and PEP. It may therefore be sensible to extend the circle of contacts per index patient with more distant contacts when the NCDR is declining. Also, screening and providing SDR to an entire community or island may be considered to reduce NCDR in a relatively shorter period, as demonstrated in a recent modelling study.[18]

In this study, we modeled subnational administrative units of seven countries. As a result, the predicted reduction in new cases is setting specific and may therefore not be generalizable to the whole country. However, we observe a similar pattern of the impact of the LPEP program across all modeled areas, i.e. an initial increase in number of new cases detected followed by a shaper decline. We may expect a similar pattern in other subnational administrative units if the LPEP program would be implemented nationwide.

The quality of the routinely reported data remains a concern in this study. The reported NCDRs are known to underestimate the true number of new cases and to reflect operational changes over time. Some of these changes are reported, but many remain unknown.[29] Therefore, we had to estimate detection delays under passive case detection conditions based on the changes observed in the NCDR, assuming that any change corresponds to an improvement in control (i.e. shorter detection delay). This assumption may, however, not hold everywhere (i.e. areas with decreased control efforts).[12] Long detection delays in our model reflect a situation of high number of undiagnosed cases in the modelled population; an assumption that cannot be validated because the true number of cases is unknown. In addition, some LPEP areas have limited or even no historical trend data of the NCDR. In Myanmar, for example, we used the (relatively low) national NCDR trend to predict the impact of LPEP in a relatively high endemic area. This likely resulted in an underestimation of the impact of the LPEP program in this area. Finally, our predictions are based on a continuation of the LPEP program as implemented in each area. Any changes in the deployment of this strategy have not been considered in the predictions.

## Conclusion

Our model shows that the LPEP program, if continued, could further reduce the leprosy case detection rates significantly and prevent many new leprosy cases. Significant reductions in NCDR are mainly noticeable after some years. The extent of the impact of the LPEP program depends primarily on case finding efforts and the number contacts per index patient included. Contact tracing and screening, combined with SDR, has the potential to accelerate NCDR reduction, and might therefore be a promising strategy to interrupt of *M*. *leprae* transmission. As the model reveals increased benefits over time, we advise to include contact tracing and screening together with SDR into routine leprosy control programmes.

## Supporting information

S1 Text Model description. Section A: Demography; Section B: Leprosy. Table A: Demographic data to quantify the model; Table B: Parameters describing household processes; Table C: Parameters to quantify natural history of infection with M. leprae; Table D: Parameters to quantify transmission; Table E: Parameters to quantify treatment and control; Table F: Parameters to quantify the LPEP program; Table G: Overview of calibrated parameters; Table H: Passive case detection delays in years; Table I: Predicted trend of new case detection under LPEP program and routine since start of LPEP. Fig A: Age distribution used to quantify the model; Fig B: Population size of Brazil, India, Indonesia, Myanmar, Nepal, Sri Lanka and Tanzania; Fig C: Survival curves for males and females used to quantify the model; Fig D: Fraction of males and females per age group currently married used to quantify the model; Fig E: Age specific birth rates used to quantify the model; Fig F: The observed and modelled household size distribution; Fig G: Leprosy new case detection rate by LPEP study area; Fig H: BCG coverage rates in infants; Fig I: Comparison of predicted trends with the observed new case detection rates of leprosy; Fig J: Model validation; Fig K: Comparison of predicted trends with the observed new case detection rates of leprosy; Fig L: Comparison of predicted trends with the observed new case detection rates of MB leprosy; Fig M: Comparison of predicted trends with the observed new case detection rates of leprosy during LPEP program; Fig N: Predicted long term trends of the leprosy new case detection rate under the routine programme, and combined with contact tracing only and the LPEP program, stratified by LPEP area; Fig O: Contacts received SDR by year by LPEP study area
(PDF)
