# Supplementary material for: The long-term impact of the Leprosy Post-Exposure Prophylaxis (LPEP) program on leprosy incidence: A modelling study
Source: PLoS Negl Trop Dis. 2021 Mar 31;15(3):e0009279. doi: 10.1371/journal.pntd.0009279 (PMC8011751; doi:10.1371/journal.pntd.0009279)
Supplement: S1 Text — Section A: Demography; Section B: Leprosy. Table A: Demographic data to quantify the model; Table B: Parameters describing household processes; Table C: Parameters to quantify natural history of infection with M. leprae; Table D: Parameters to quantify transmission; Table E: Parameters to quantify treatment and control; Table F: Parameters to quantify the LPEP program; Table G: Overview of calibrated parameters; Table H: Passive case detection delays in years; Table I: Predicted trend of new case detection under LPEP program and routine since start of LPEP. Fig A: Age distribution used to quantify the model; Fig B: Population size of Brazil, India, Indonesia, Myanmar, Nepal, Sri Lanka and Tanzania; Fig C: Survival curves for males and females used to quantify the model; Fig D: Fraction of males and females per age group currently married used to quantify the model; Fig E: Age specific birth rates used to quantify the model; Fig F: The observed and modelled household size distribution; Fig G: Leprosy new case detection rate by LPEP study area; Fig H: BCG coverage rates in infants; Fig I: Comparison of predicted trends with the observed new case detection rates of leprosy; Fig J: Model validation; Fig K: Comparison of predicted trends with the observed new case detection rates of leprosy; Fig L: Comparison of predicted trends with the observed new case detection rates of MB leprosy; Fig M: Comparison of predicted trends with the observed new case detection rates of leprosy during LPEP program; Fig N: Predicted long term trends of the leprosy new case detection rate under the routine programme, and combined with contact tracing only and the LPEP program, stratified by LPEP area; Fig O: Contacts received SDR by year by LPEP study area (PDF) [file pntd.0009279.s001.pdf]

S1 Model description

SIMCOLEP is a stochastic individual-based model, which models leprosy transmission in a population structured by household. It simulates the life histories of individuals, the natural history of infection with *M. leprae* and the transmission of *M. leprae* from person to person. The effects of different control strategies, including active case finding, contact screening and chemoprophylaxis, on the transmission and the leprosy incidence can be evaluated and predicted. Since its development, the model has been used in multiple applications.[1-9] A full description of the model is provided by Fischer et al. and Blok et al.[1, 4]

The model was fitted to the leprosy situation in states or districts of seven countries:

1) Alta Floresta city and region and Rondonópolis city in Mato Grosso, Araguaína and Colinas do Tocantins in Tocantins, and Petrolina city and region in Pernambuco, Brazil
2) Union Territory Dadra and Nagar Haveli, India
3) Sumenep district, Indonesia
4) Nyaung Oo, Myingyan and Tharyarwaddy Townships, Myanmar
5) Jhapa, Morang and Parsa districts, Nepal
6) Kalutara and Puttalam districts, Sri Lanka
7) Kilombero, Liwale and Nanyumbu districts, Tanzania

These subnational areas are henceforth addressed as Brazil, India, Indonesia, Nepal, Sri Lanka and Tanzania.

A. Demography

The model was quantified with demographic data including population growth rate, fertility and survival rates, age and sex distribution, the fraction of married population per age group and household size distribution (Table A).

Table A. Demographic data to quantify the model

Country	Parameter	Level	Years	Source
Brazil	Population size	Country<br>District	1872-1991,<br>1991-2010	IBGE[10-13]
	Survival rates	Country	1965, 1975,<br>1990, 2000, 2010	NRC[14]<br>WHO[15]
	Age-specific fertility rates	Country	1980, 1991, 2000, 2010	IBGE[11-13, 16]
	Age distribution	Country	2010	IBGE[11]
	Fraction married	Country	1980, 1991, 2000, 2010	IBGE[11-13, 16]
	Household size distribution	Country	2010	IBGE[11]
India	Population size	Country	1901-2011	Census India[17]
	Survival rates	Country	1995, 1999, 2003, 2007, 2011	Census India[18]
	Age-specific fertility rates	District	1990, 1993, 1996, 1999, 2006, 2011	Census India[18]
	Age distribution	District	2011	Census India[19]
	Fraction married	District	1991, 2001, 2011	Census India[19]
	Household size distribution	District	2011	Census India[19]
Indonesia	Population size	Country	1900-1951<br>1971-2010	Nitisastro[20]<br>Statistics Indonesia[21]
	Survival rates	Country	2000, 2010	WHO[22]
	Age-specific fertility rates	Country	2012	Statistics Indonesia[23]
	Age distribution	Country	2012	Statistics Indonesia[23]
	Fraction married	Country	2010	UN data[24]
	Household size distribution	Country	2012	Statistics Indonesia[23]
Myanmar	Population size	Country	1950-2010	UN[25]
	Survival rates	Country	2000, 2010	WHO[26]
	Age-specific fertility rates	Country	2014	Myanmar Census[27]

	Age distribution	Country	2014	Myanmar Census[27]
	Fraction married	Country	2014	Myanmar Census[27]
	Household size distribution	Country	2014	Myanmar Census[27]
Nepal	Population size	Country	1911-2011	CBS[28]
	Survival rates	Country	2000, 2010	WHO[29]
	Age-specific fertility rates	Country	1996, 2001, 2006, 2011	DHS[30]
	Age distribution	District	2011	CBS[28]
	Fraction married	Country	2011	CBS[28]
	Household size distribution	District	2011	CBS[28]
Sri Lanka	Population size	Country	1901-2012	Dept. Census and Statistics[31]
	Survival rates	Country	2000, 2010	WHO[32]
	Age-specific fertility rates	Country	2006	DHS[33]
	Age distribution	Country	2006	DHS[33]
	Fraction married	Country	2012	Dept. Census and Statistics[34]
	Household size distribution	Country	2006	DHS[33]
Tanzania	Population size	Country<br>District	1967-2002,<br>2003-2013	Agwanda et al.[35]<br>LPEP database
	Survival rates	Country	2000, 2010	WHO[36]
	Age-specific fertility rates	Country	1996, 1999, 2004, 2010	DHS[37]
	Age distribution	Country	2012	National Bureau of Statistics[38]
	Fraction married	Country	2012	National Bureau of Statistics[39]
	Household size distribution	Country	2010	DHS[37]

1. Initial population

The initial population was set to 20,000 individuals in Brazil, India, Indonesia, Nepal and Tanzania. In Myanmar and Sri Lanka, we increased the starting population to 50,000 individuals, because of low leprosy new case detection rates. The initial population follows the observed age distribution (Fig A) and the sex ratio was set to 50%.

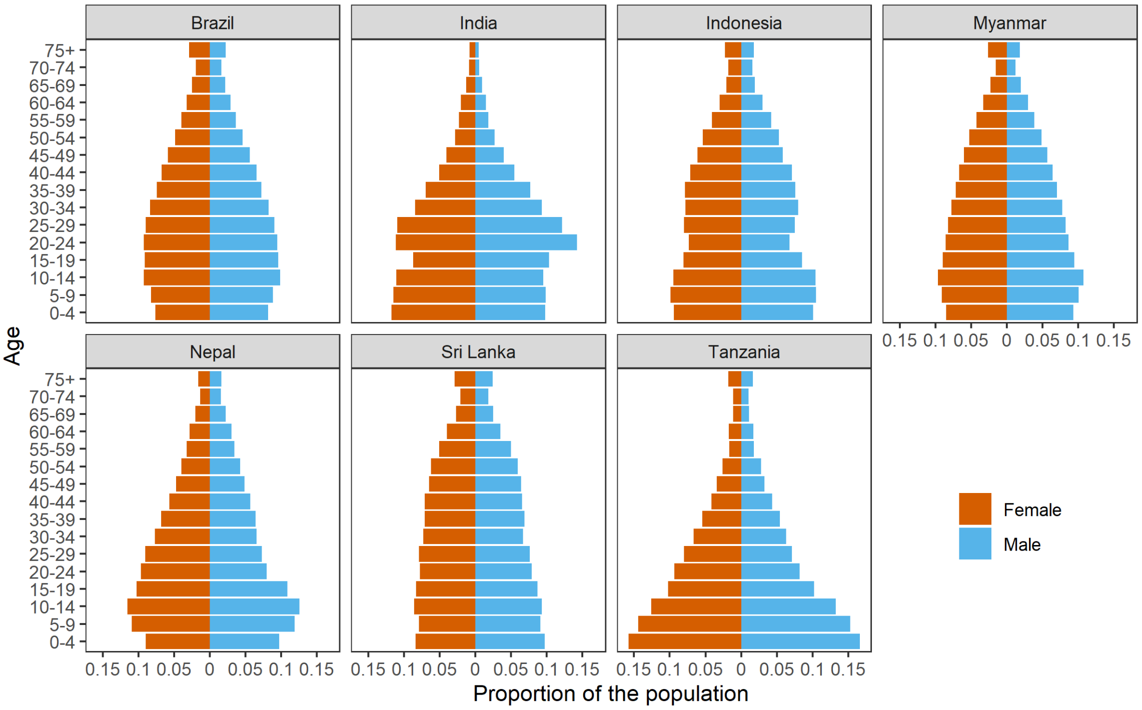

Fig A. Age distribution used to quantify the model.

2. Population growth

During the simulation, the population grows with a time-dependent growth rate (Fig B). In 2015, the simulated population size was 407,000 in Brazil, 108,000 in India, 127,000 in Indonesia, 148,000 in Myanmar, 99,000 in Nepal, 290,000 in Sri Lanka and 63,000 in Tanzania.

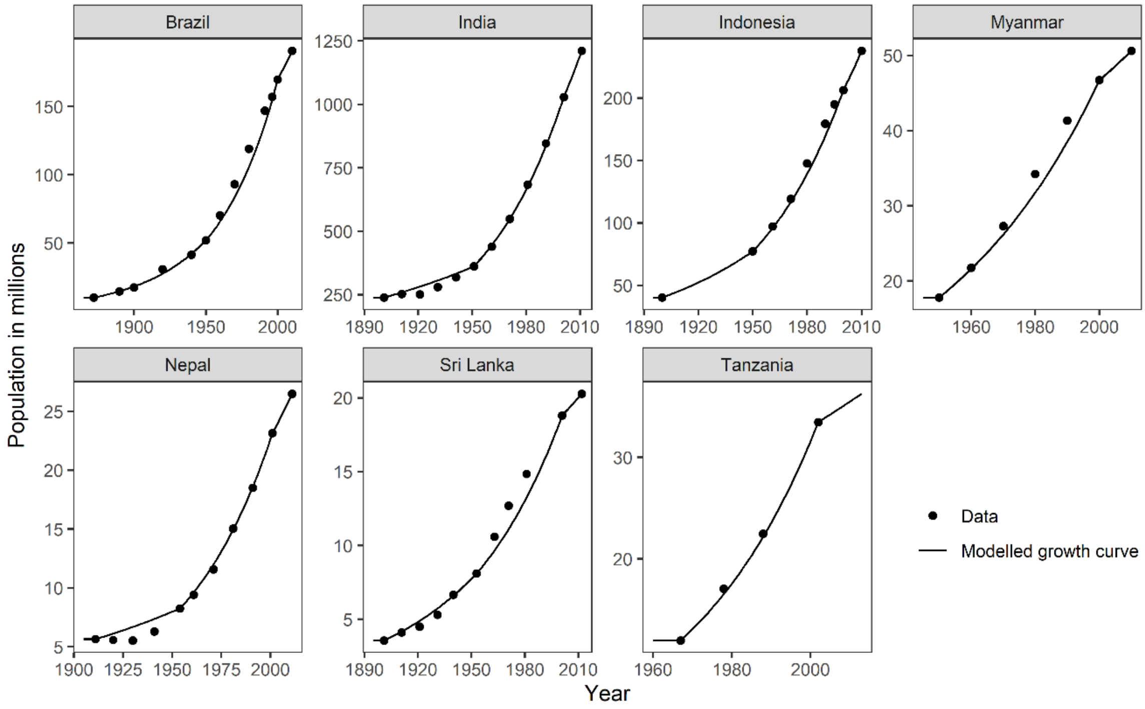

Fig B. Population size of Brazil, India, Indonesia, Myanmar, Nepal, Sri Lanka and Tanzania. The solid line is the exponential growth curve used as input for the model. The data points are population estimates. Annual growth rates: 1) Brazil: 0.021 (1872-1950); 0.024 (1950-2000); 0.012 (2000-2010)[10-13]; 2) India: 0.008 (1901-1951); 0.021 (1951-2001); 0.016 (2001-2011)[17]; 3) Indonesia: 0.013 (1900-1950); 0.020 (1950-2000); 0.014 (2000-2010)[20, 21]; 4) Myanmar: 0.019 (1950-2000); 0.008 (2000-2010) [25]; 5) Nepal: 0.009 (1911-1954); 0.022 (1954-2001); 0.013 (2001-2011) [28]; 6) Sri Lanka: 0.016 (1901-1953); 0.018 (1953-2001); 0.007 (2001-2012) [31]; 7) Tanzania: 0.029 (1967-2003); 0.007 (2003-2013) [35].

3. Birth and Death

The number of new births is determined by the difference of the current population size and the expected population size accounting for population growth. At birth, a new individual is created and the age of death is determined by sex-dependent survival curves (Fig C).

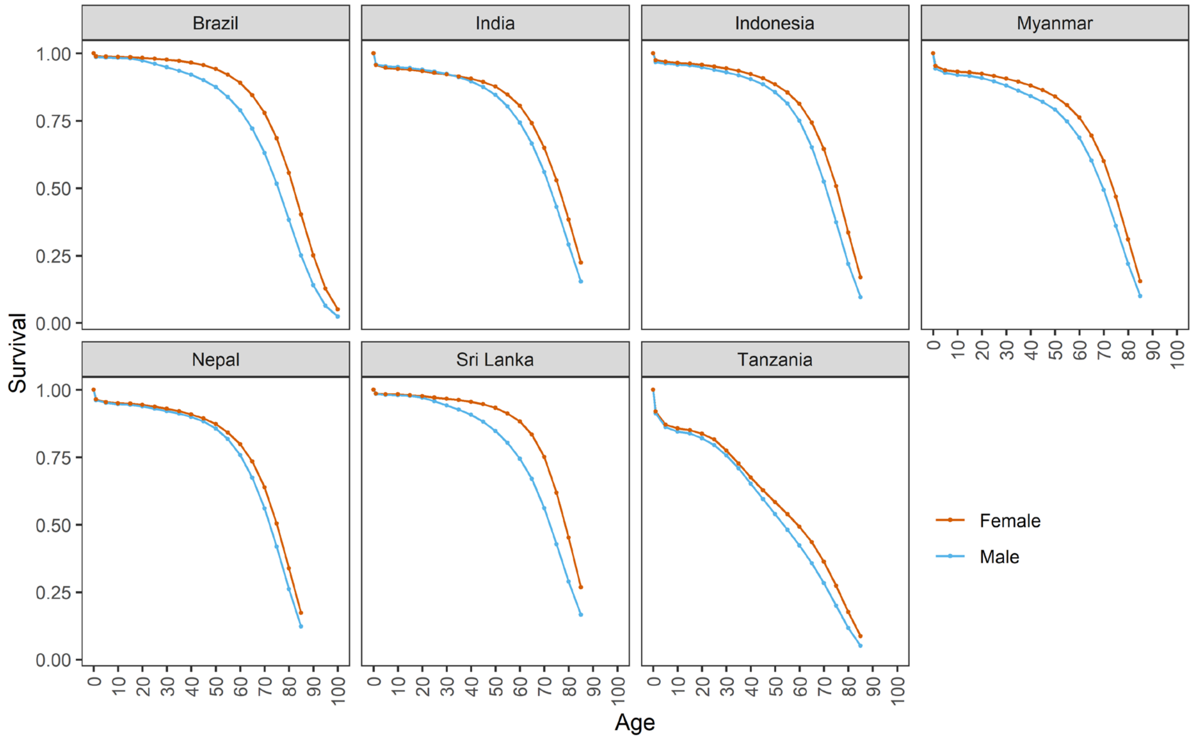

Fig C. Survival curves for males and females used to quantify the model. Data shown from Brazil (2013)[14, 15], India (2011)[18], Indonesia (2010)[22], Myanmar (2010)[26], Nepal (2010)[29], Sri Lanka (2010)[32] and Tanzania (2010)[36].

4. Household size distribution

The model was fitted to the observed household size distribution in each LPEP study area. In the model, household formation, changes and dissolution are determined by three processes: 1) random movements by individuals; 2) marriage or relationships and 3) newborns.

Only a fraction of the population moves randomly. Based on previous modeling studies, we assume that the age of random movement is chosen randomly from a uniform distribution between 12 and 22 years of age, representing movement out of parental household or movements to other family members. This assumption was based on previous modeling work.[4] A small fraction will create their own household, while the remainder will move into an existing household. The size of the household to move to is randomly determined following a triangular distribution. At marriage, a fraction of couples creates a new household, while the other couples will become a member of the household of the male. In the latter case, the household will split up after 12 years on average (exponential distributed). After the death of a married person, the surviving spouse is again a candidate for marriage again. If the surviving spouse is left alone, he/she will move to the household of one his/her children.

In the model males and females can be coupled such that the proportion of married males and females in each age group matches data (Fig D). Newborns are placed in a household of a married female. The married female (i.e. mother) is randomly selected based on the age specific birth rates (Fig E).

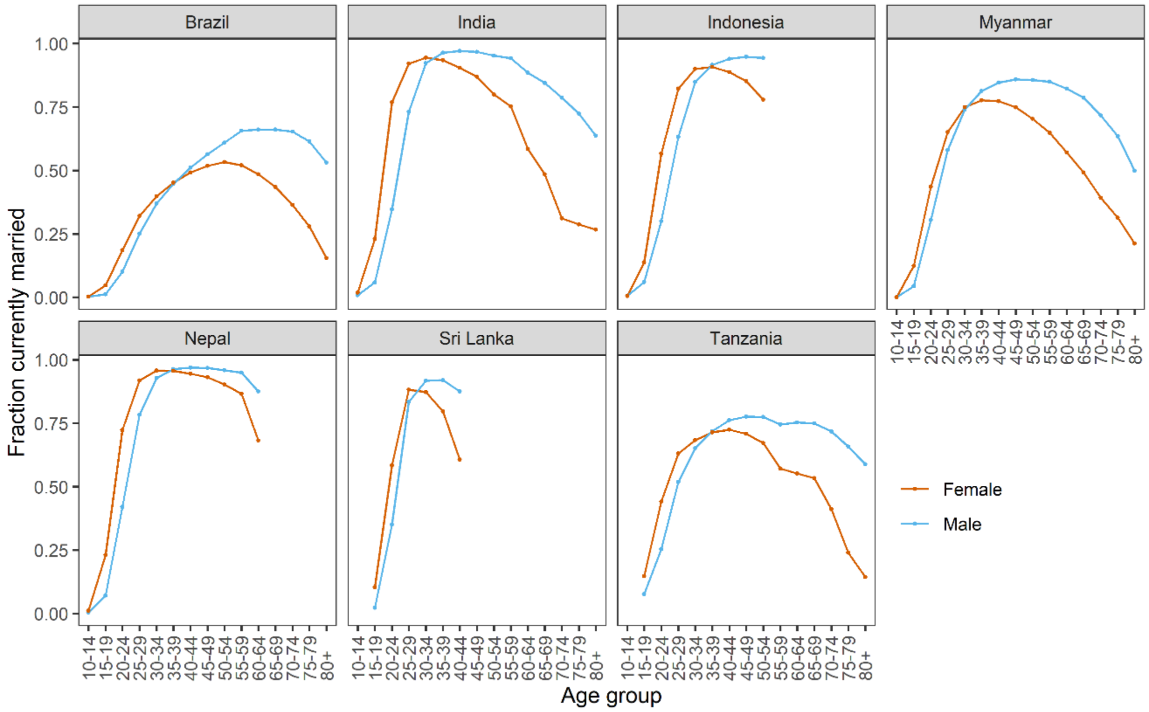

Fig D. Fraction of males and females per age group currently married used to quantify the model. Data from Brazil (2010), India (2011), Indonesia (2012), Myanmar (2014), Nepal (2011), Sri Lanka (2012) and Tanzania (2012).

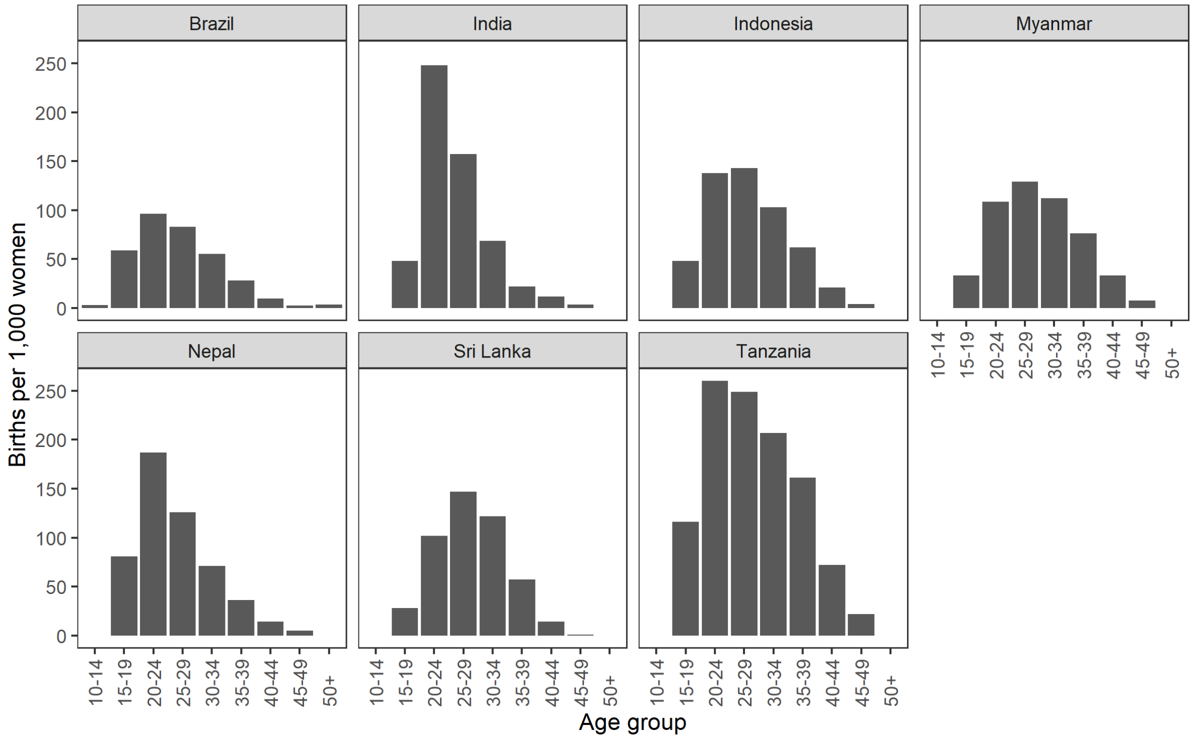

Fig E. Age specific birth rates used to quantify the model. Data from Brazil (2010)[11-13, 16], India (2011)[18], Indonesia (2012)[23], Myanmar (2014)[27], Nepal (2011)[30], Sri Lanka (2006)[33] and Tanzania (2010)[37].

The parameters that determine household processes include: fraction random movement, fraction that creates their own household, distribution of household size to move to, fraction of married couple creating own household, duration of splitting a married household from parental household and the fraction of single widows that moving to children (Table B). Only those parameters that were essential to replicate observed household distribution were calibrated. Optimal values of calibrated parameters were derived through a grid search. The goodness-of-fit of the distribution of household size was evaluated by a Chi-square test. Fig F shows the results of the calibration. For all countries, there is no significant difference between the simulated and observed distribution.

Table B. Parameters describing household processes

Parameters	Brazil	India	Indonesia	Myanmar	Nepal	Sri Lanka	Tanzania
Fraction random movement	0.80 [a]	0.71 [a]	0.76 [a]	0.73 [a]	0.29 [a]	0.72 [a]	0.51 [a]
Fraction creates own household	0.15 [a]	0 [b]	0 [b]	0 [b]	0 [b]	0 [b]	0.07 [a]
Household size to move to (Triangular distribution)	(0, 4, 1) [a]	(0, 4, 3) [a]	(0, 4, 2) [a]	(0, 4, 2) [a]	(0, 5, 3) [a]	(0, 4, 2) [a]	(0, 6, 1) [a]
Fraction of married couple creating own household	0.85 [a]	0.25 [c]	0.25 [c]	0.25 [c]	0.25 [c]	0.20 [a]	0.25 [c]
Time until splitting of a married household from parental household (Exponential distribution)	12 [c]	12 [c]	12 [c]	12 [c]	12 [c]	12 [c]	12 [c]
Fraction single widow(er)s moving back to children	1.0 [b]	1.0 [b]	1.0 [b]	1.0 [b]	1.0 [b]	1.0 [b]	1.0 [b]

[a] Calibrated; [b] Assumption; [c] Based on Fischer et al.[4]

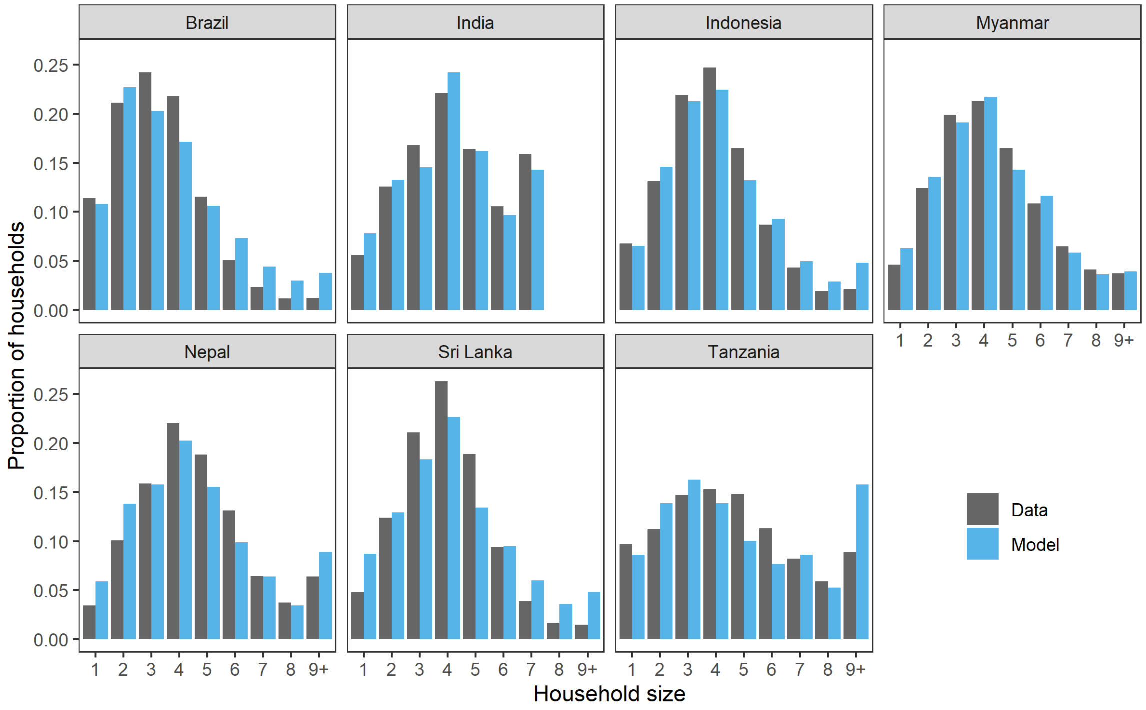

Fig F. The observed and modelled household size distribution. There is no significant difference between data and modeled distribution: Brazil (p = 0.56, χ^2- test), India (p = 0.96, χ^2- test), Indonesia (p = 0.92, χ^2- test), Myanmar (p = 0.99, χ^2- test), Nepal (p = 0.79, χ^2- test), Sri Lanka (p = 0.34, χ^2- test), Tanzania (p = 0.40, χ^2- test).

B. Leprosy

After the model was fitted to the demographics of each LPEP study area, the new case detection rate (NCDR) in the model was fitted to the data. Area specific historical NCDR data and MB proportion was available for all LPEP area, except for Myanmar for which we used country level NCDR data (Fig G). The leprosy module includes the natural history of infection, transmission, treatment and control.

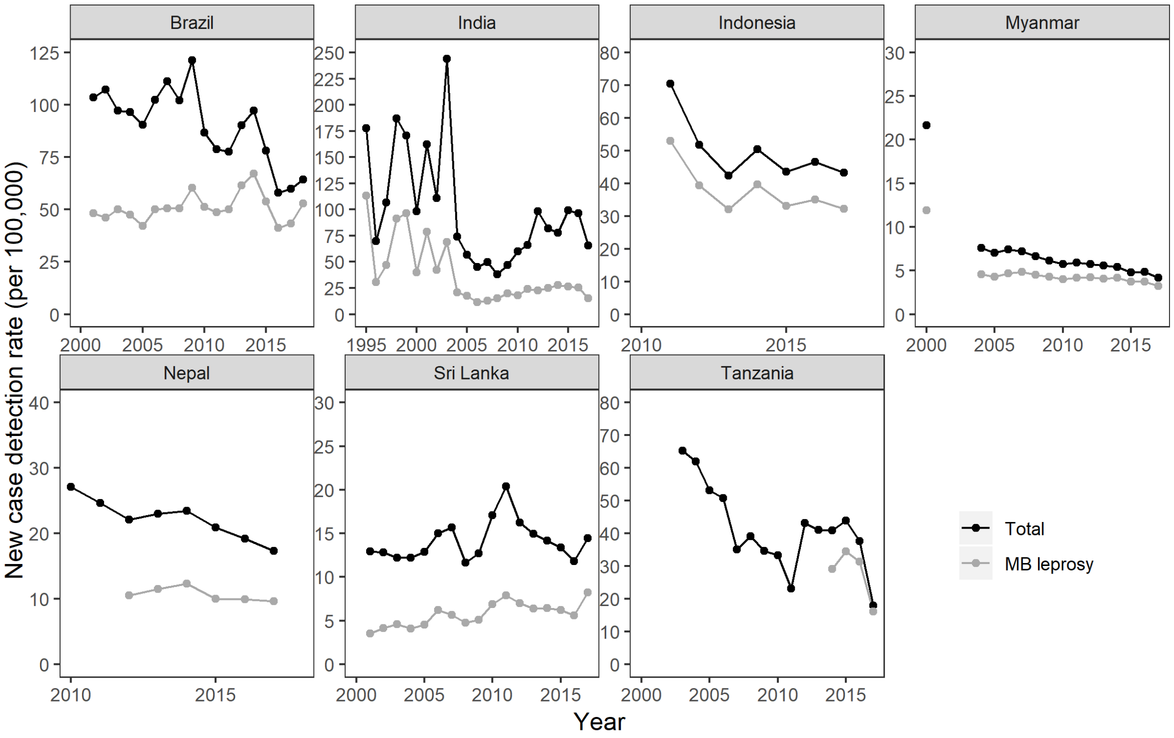

Fig G. Leprosy new case detection rate by LPEP study area.

1. Natural history of infection

The natural history of leprosy is modelled following Meima et al.[40] Table C provides an overview of the parameters used to quantify the natural history of infection.

Table C. Parameters to quantify natural history of infection with *M. leprae*

Parameters	Value	Source
Proportion susceptible	20% [a]	Assumption
MB / PB ratio	26 / 76	NLEP India [41]
PB subclinical duration mean	4.2 years; SD =1.9 (gamma distributed)	Fischer et al. & Fine[4, 42]
PB self-healing rate	20% per year	Fischer et al. & Sirumban et al. [4, 43]
MB subclinical duration mean	11.1 years; SD = 5.0 (gamma distributed)	Fischer et al. & Fine[4, 42]

[a] Previous modeling showed that assuming 5%, 10% and 20% did not provide significant different results (Fischer et al.[4])

2. Transmission

Two transmission processes are modelled separately: transmission in the general population and within-household. Transmission in the general population is made indiscriminately to individuals within and outside the household, while the within-household transmission takes place within the household of an infectious individual. The previously calibrated within-household contact rate of 0.98 was used assuming that this would not differ across countries and districts. The probability of transmission is determined by the contact rate and the infectivity function (Table D).

Table D. Parameters to quantify transmission

Parameters	Value	Source
Contact rate		
General population	India: (3, 7)<br>Brazil: (0.5, 2)<br>Indonesia: (0.1, 0.6)<br>Tanzania: (0.1, 0.4)<br>Nepal: (0.4, 0.8)<br>Sri Lanka: (0.4, 1.2)<br>Myanmar: (0.2, 0.3)	Calibrated [a]
Within households	0.98	Fischer et al.[4]
Infectivity function		Meima et al.[44]
PB	0	
Asymptomatic MB	Linear from 0 to 1	
Symptomatic MB	1	

[a] Calibrated to match modelled leprosy new case detection rate trend to data.

3. Treatment and control

Treatment with MDT is given to each patient that is diagnosed. After treatment the patient is considered not to be infectious anymore. Relapse after treatment for both PB and MB leprosy may occur. The model also mimics the leprosy control program, which includes passive cased detection and active case surveys or contact tracing. Passive case detection is reflected by passive case detection delay, which was calibrated to match the observed historic NCDR trend. Based on the data and the information through national leprosy reports, we identified years of operational changes and surveys (Table E). We assumed that at times of operational changes the passive case detection delays would improve. We used a logistic function to determine changes of passive case detection delays over time.

$$DD(t) = \left(\frac{max - min}{1 + e^{b\cdot(t-mid)}}\right) + min$$

The minimum detection delay was set to 2 years. The maximum years detection delay (max), the midpoint of the function (mid) and the slope (b) were calibrated. Surveys were included if the was evident from available report. The coverage was calibrated to match observed NCDR in that particular year. The model also includes the protective effect of BCG vaccination in infants. BCG coverage rates in infants from 1980 onwards were used to quantify the model (Fig H).

Table E. Parameters to quantify treatment and control

Parameters	Value	Source
**Treatment**		
MDT use	1990 onwards	
MDT relapse rate	0.01 per year<br>To MB: 90%<br>To PB: 10%	Meima et al. & Becxbleumink[45, 46]
**Control**		
Passive case detection delays		
Years of improved detection delay	India: 1995, 1998, 1999, 2001, 2011, 2012<br>Brazil: 2000<br>Indonesia: 2010<br>Tanzania: 2002<br>Nepal: 2008<br>Sri Lanka: 2010<br>Myanmar: 199	Based on leprosy data
Survey		
Year	India: 2002	Based on data from NLEP India [41]
BCG protection	60%	Schuring et al.[47]

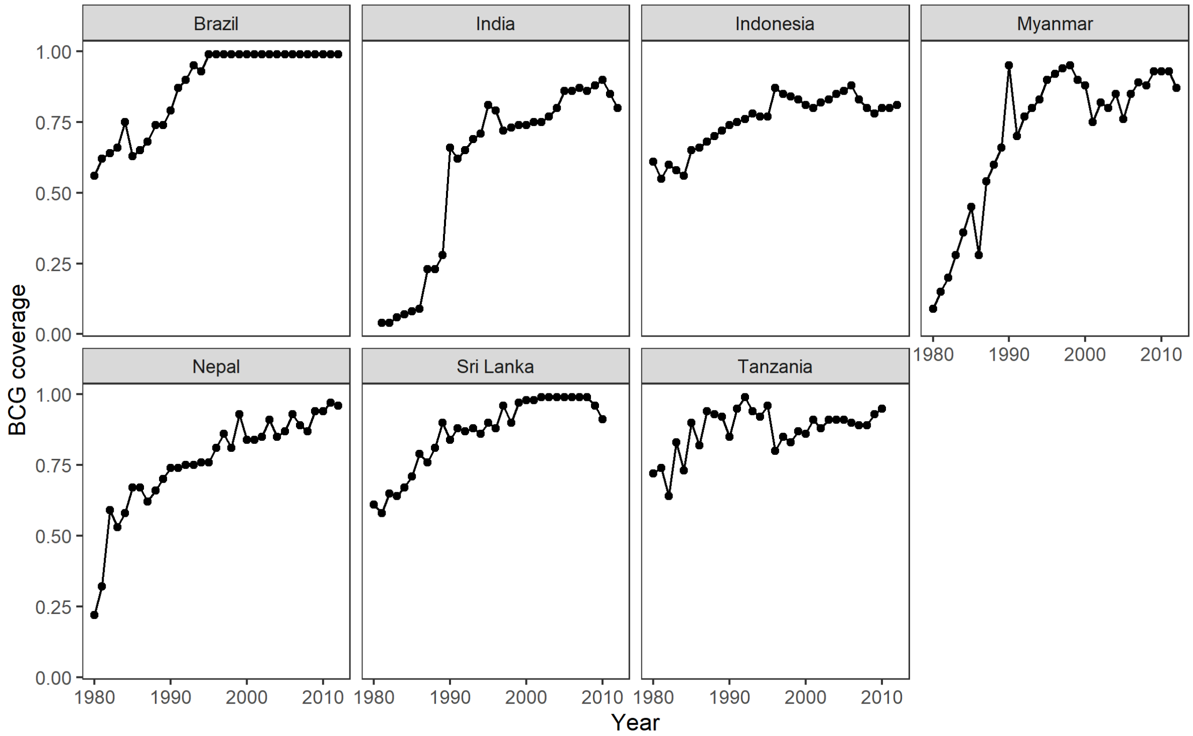

Fig H. BCG coverage rates in infants. Source: UN data[48] and DHS[37]

4. LPEP program

In the model, household contacts and neighbours of diagnosed patients were traced and screened. Only contacts without clinical leprosy and contacts, who had not taken SDR before, were given SDR.[49] Table F provides an overview of the LPEP program parameters, which are too large extent obtained from the LPEP program data.

Table F. Parameters to quantify the LPEP program

Parameters	Value	Source
**Contact tracing and screening**		
Start	2015 (2016 in Brazil and Sri Lanka)	LPEP program data
Probability of detecting clinical leprosy	0.9	Assumption
Number of contacts screened per index patient	India: 26 contacts<br>Brazil: 11<br>Indonesia: 36<br>Tanzania: 9 (only household)<br>Nepal: 23<br>Sri Lanka: 1 (only household)<br>Myanmar: 18	LPEP program data
Retrospective contact tracing (years prior to start)	2 years (India and Nepal)<br>1 year (Brazil, Tanzania, Sri Lanka, Myanmar)<br>0.5 year (Indonesia)	LPEP program data[49]
Coverage	99%	LPEP program data
**SDR**		
Effectiveness		Moet et al.[50]
Household contacts	50%	
Other contacts	70%	

5. Model calibration

For the calibration, we randomly drew parameter values from uniform distributions with intervals wide enough to capture all possible values that could produce a good fit. The model was run with these parameter values, which were accepted if the fit was good. Table G present all calibrated parameters to fit the historic trend of NCDR in each LPEP study area.

Table G. Overview of calibrated parameters

Parameters	Brazil	India	Indonesia	Myanmar	Nepal	Sri Lanka	Tanzania
Contact rate in the general population	(0.5, 2)	(3, 7)	(0.1, 0.6)	(0.2, 0.3)	(0.4, 0.8)	(0.4, 1.2)	(0.1, 0.4)
Detection delay							
min	2	2	2	2	2	2	2
Max	(20, 40)	(20, 40)	(20, 40)	(20, 40)	(20, 40)	(20, 40)	(20, 40)
b (slope)	(0.05, 2)	(0.05, 2)	(0.05, 2)	(0.05, 2)	(0.05, 2)	(0.05, 2)	(0.05, 2)
mid	(0, 6)	(0, 9)	(0, 4)	(0, 4)	(0, 3)	(0, 3)	(0, 3)
Survey coverage	-	(0.05, 0.2)	-	-	-	-	-

The goodness of fit was assessed using a log-likelihood assuming a Poisson distribution. We repeated this until we had 1,000 parameter combinations. A parameter combination was accepted when the log-likelihood did not deviate from the maximum log-likelihood more than 1.5 times. Uncertainty intervals, which reflect uncertainty in the parameter values, were calculated by discarding the 2.5% highest and lowest values. The model was first calibrated using NCDR data until 2012 in order to evaluate the ability of the model to forecast the data points in 2013 and 2014 (Fig I and J).

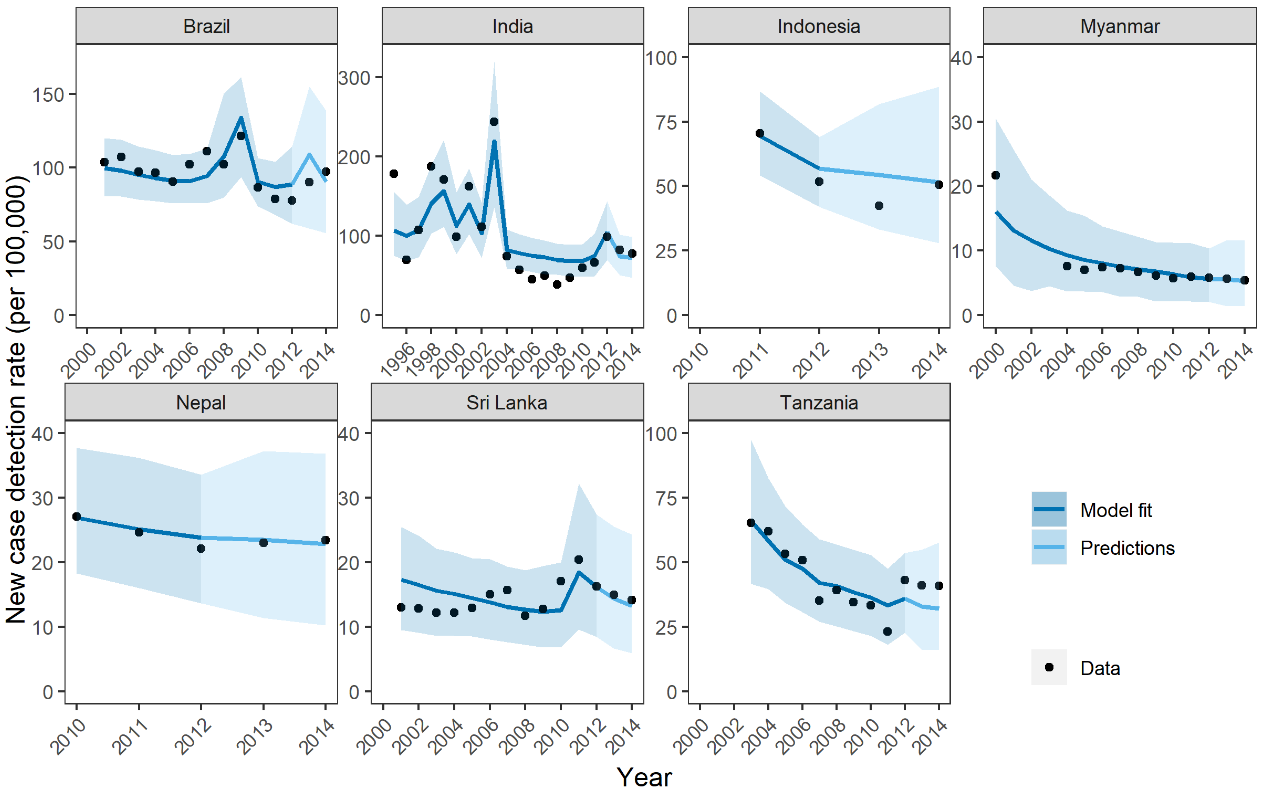

Fig I. Comparison of predicted trends with the observed new case detection rates of leprosy. Data are represented by black dots. Solid blue lines represent mean estimates and the shaded area the 95% prediction intervals (shaded areas). The dark blue lines and shaded area represent result from model fitting and the light blue lines and shaded areas the model predictions. The model was fitted using data until 2012. Predictions were made for 2013 and 2014.

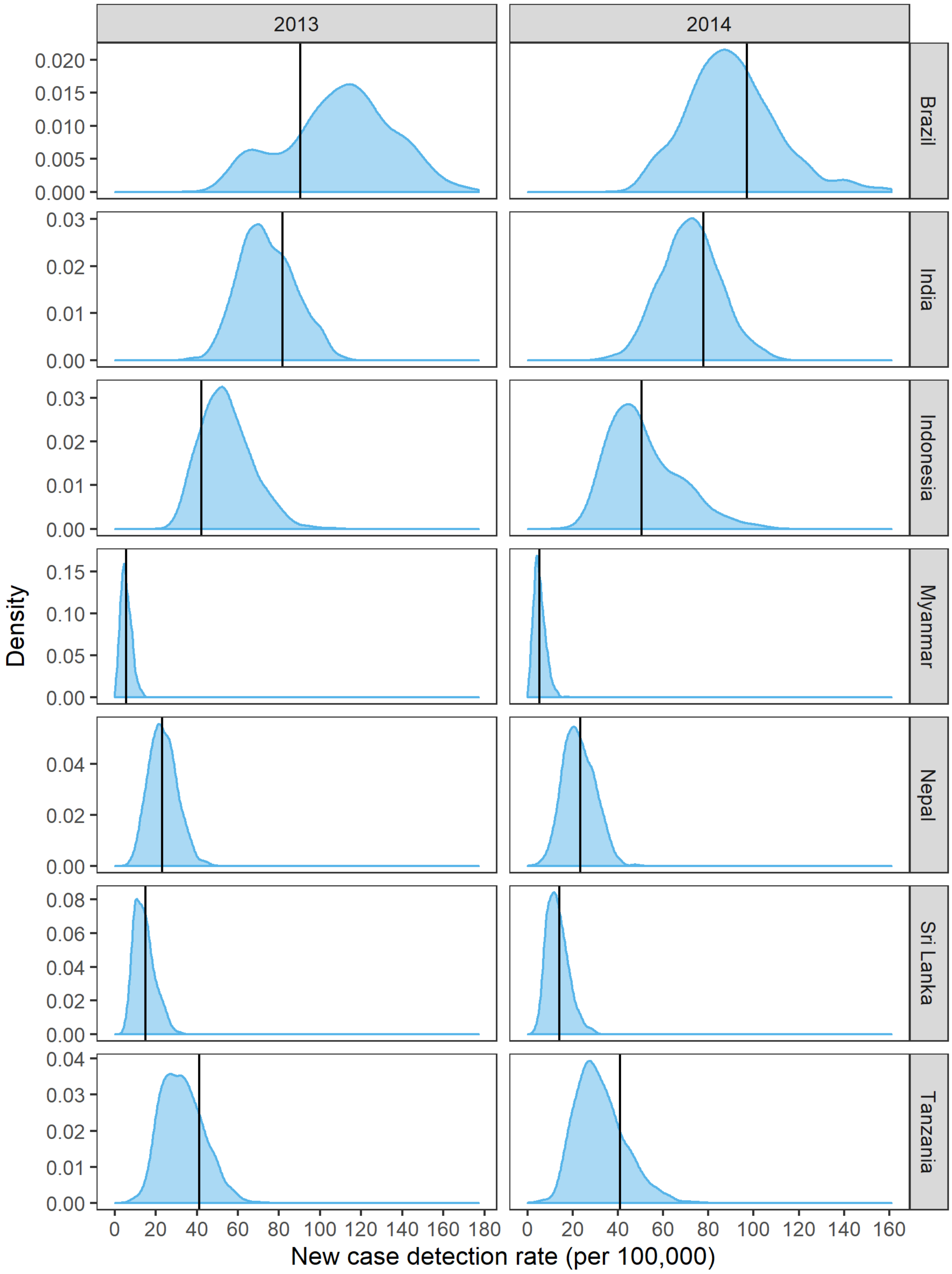

Fig J. Model validation. Distribution of forecasted numbers of new cases of leprosy in 2013-2014 by LPEP study area. The observed value for each area-year combination is indicated by a vertical black line.

After evaluating the short-term predictions, we fitted the model to the data until 2014 (Fig K). We excluded the data of 2015 and beyond because in these years the LPEP program was implemented. The corresponding calibrated passive detection delays are presented in Table H. Fig L shows the associated modelled MB leprosy detection rate trend compared to the data by study area. The MB detection rates are influenced by the assumed initial MB proportion among leprosy cases and the detection delays. With longer the delays more PB cases are missed. The initial MB proportion was set as follows: Brazil (40%), India (15%), Indonesia (70%), Tanzania (60%), Nepal (30%), Sri Lanka (20%), and Myanmar (40%).

The fitted model was used to make predictions of the routine program (i.e., counterfactual) as presented in the main results.

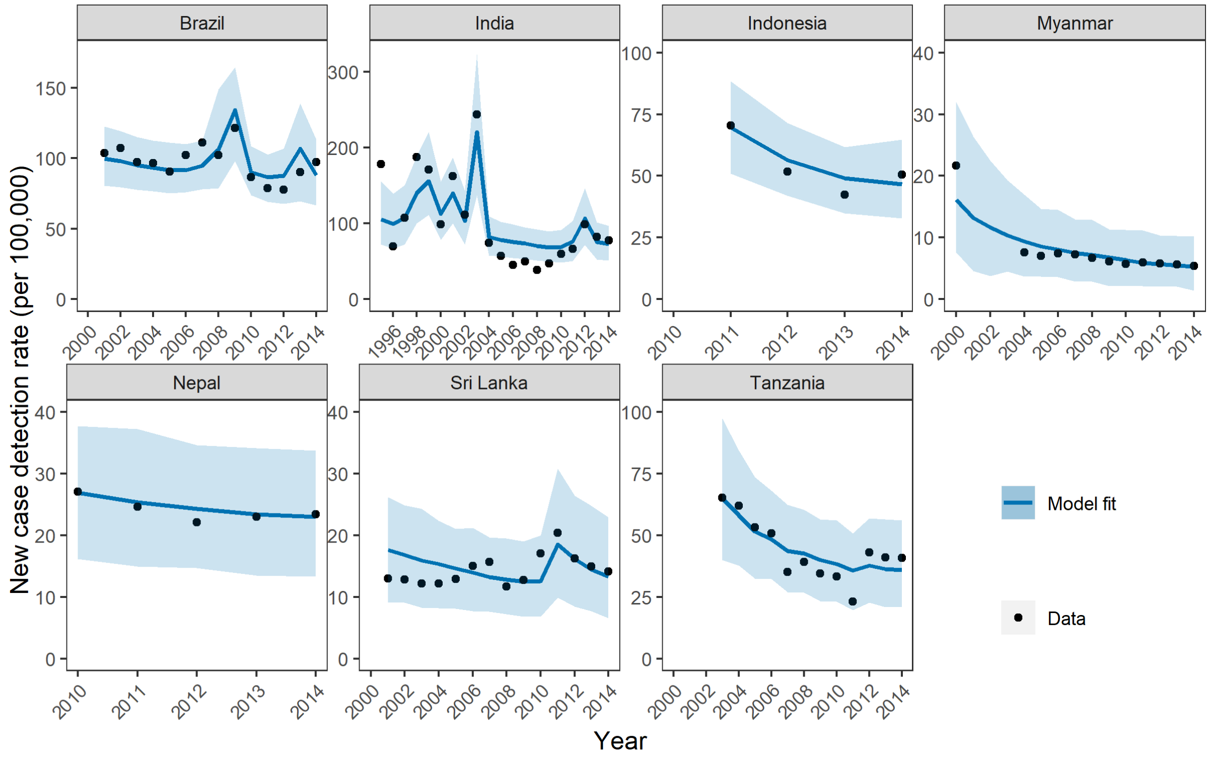

Fig K. Comparison of predicted trends with the observed new case detection rates of leprosy. Data are represented by black dots. Solid blue lines represent mean estimates and the shaded area the 95% prediction intervals (shaded areas). The model was fitted using data until 2014.

Table H. Passive case detection delays in years

Country	Mean	SD
Brazil	12.0	5.1
India	6.0	3.1
Indonesia	4.2	4.2
Myanmar	8.4	6.8
Nepal	12.9	6.7
Sri Lanka	9.6	5.7
Tanzania	7.7	5.2

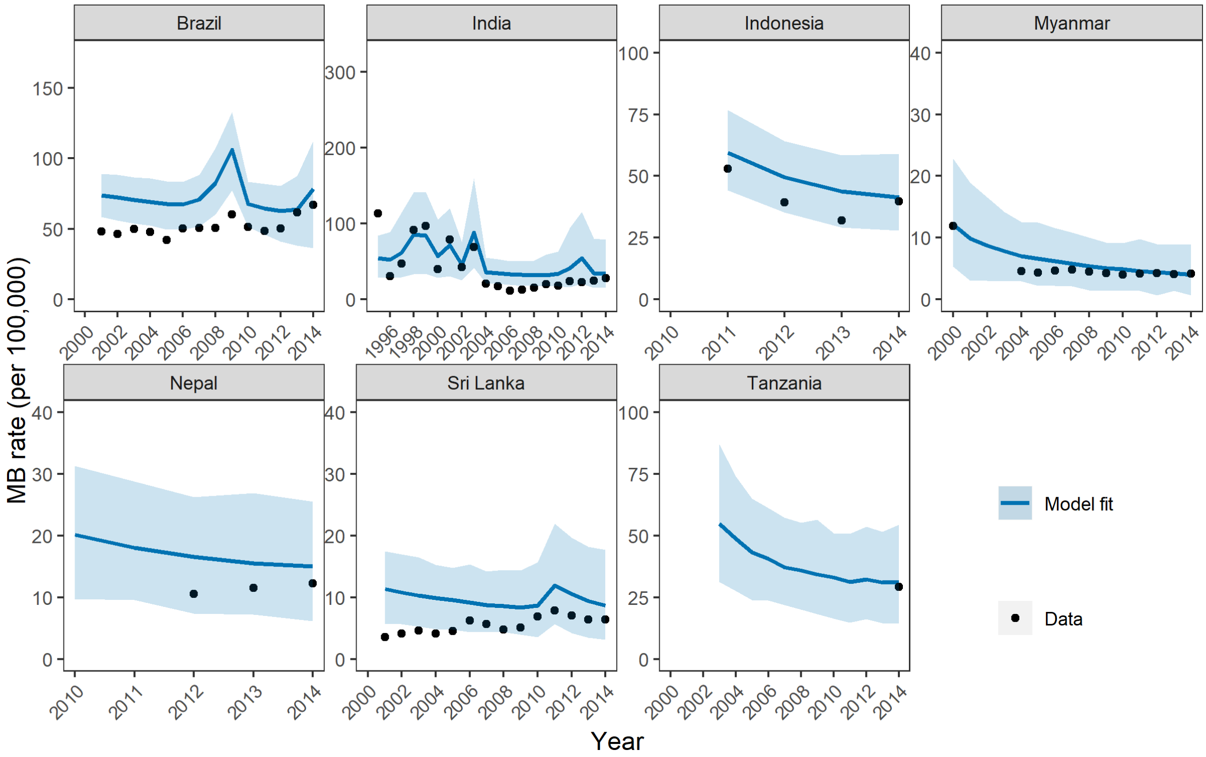

Fig L. Comparison of predicted trends with the observed new case detection rates of MB leprosy. Data are represented by black dots. Solid blue lines represent mean estimates and the shaded area the 95% prediction intervals (shaded areas).

Finally, the predictions of the LPEP scenario were validated against the new case detection rate data obtained during the LPEP program. The LPEP scenario is based on the fitted model (Fig K) and the LPEP parameter settings (Table F). Fig M shows the how the modelled LPEP scenario fits to the LPEP data. Table I provides the predicted trends of new case detection.

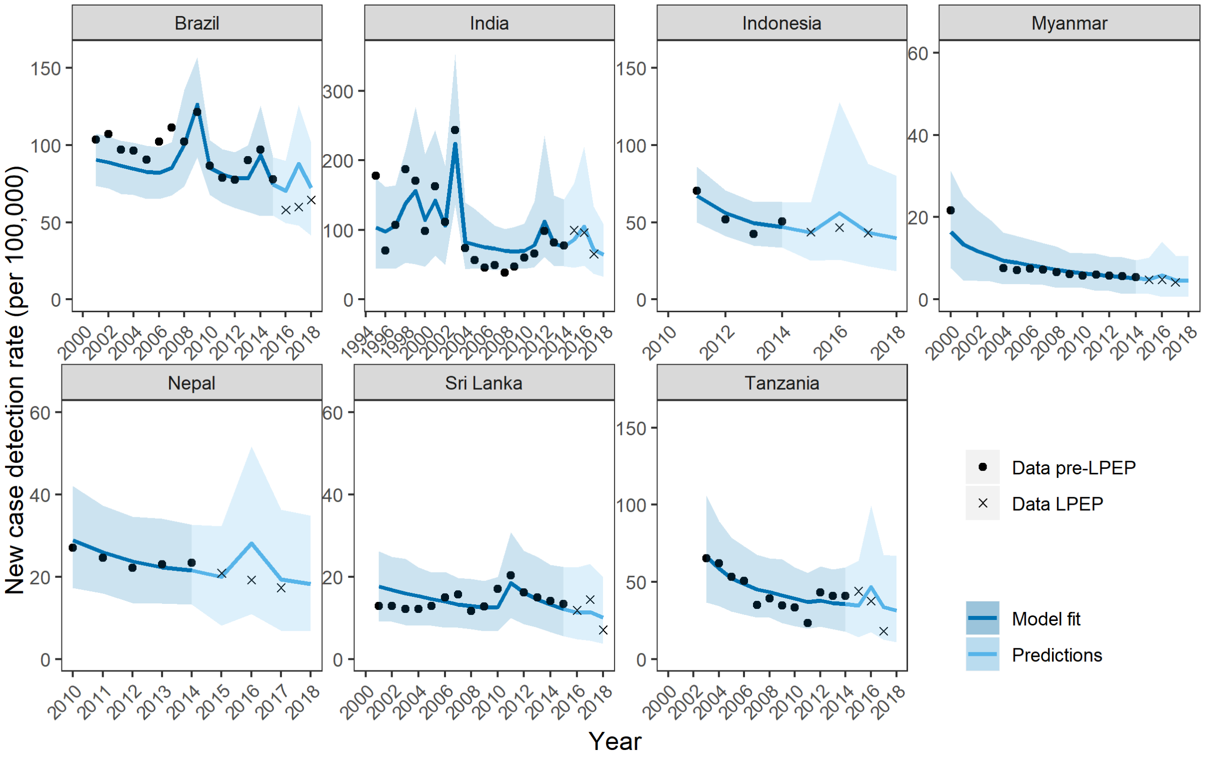

Fig M. Comparison of predicted trends with the observed new case detection rates of leprosy during LPEP program. Data pre-LPEP are represented by black dots, and data during LPEP by crosses. Solid blue lines represent mean estimates and the shaded area the 95% prediction intervals (shaded areas). The dark blue lines and

Table I. Predicted trend of new case detection under LPEP program and routine since start of LPEP

Year	Program	Brazil [a]	India	Indonesia	Myanmar	Nepal	Sri Lanka [a]	Tanzania
2016	LPEP	-	443 (203, 929)	463 (212, 1058)	171 (19, 407)	710 (276, 1307)	-	269 (100, 575)
2017	LPEP	902 (492, 1292)	306 (157, 576)	362 (180, 737)	136 (19, 310)	497 (176, 930)	236 (91, 478)	196 (73, 393)
2020	LPEP	680 (349, 994)	245 (127, 384)	301 (134, 641)	115 (0, 310)	416 (151, 830)	186 (63, 387)	161 (46, 365)
2025	LPEP	563 (241, 903)	194 (88, 315)	230 (90, 526)	88 (0, 271)	331 (50, 704)	143 (42, 324)	120 (18, 310)
2030	LPEP	475 (172, 797)	160 (65, 269)	181 (51, 468)	65 (0, 232)	249 (50, 628)	111 (21, 281)	93 (9, 265)
2035	LPEP	400 (128, 716)	141 (46, 257)	147 (32, 423)	50 (0, 194)	203 (25, 503)	85 (14, 239)	71 (9, 228)
2040	LPEP	347 (98, 660)	126 (38, 242)	117 (19, 372)	37 (0, 174)	158 (0, 477)	66 (7, 197)	54 (0, 173)
2016	Routine	-	313 (180, 637)	337 (186, 500)	134 (19, 291)	474 (226, 804)	-	173 (73, 292)
2017	Routine	692 (441, 908)	292 (165, 549)	325 (180, 494)	125 (19, 271)	449 (176, 779)	216 (91, 422)	169 (73, 301)
2020	Routine	630 (357, 869)	250 (138, 411)	293 (141, 500)	109 (19, 252)	397 (151, 729)	181 (63, 366)	152 (55, 292)
2025	Routine	553 (266, 820)	211 (104, 319)	250 (109, 462)	88 (0, 232)	331 (75, 679)	141 (42, 309)	127 (37, 274)
2030	Routine	485 (199, 765)	184 (81, 288)	214 (77, 430)	68 (0, 213)	272 (75, 578)	113 (28, 281)	105 (18, 247)
2035	Routine	432 (162, 726)	167 (65, 280)	186 (58, 430)	56 (0, 194)	222 (25, 553)	87 (14, 232)	85 (9, 228)
2040	Routine	386 (121, 694)	157 (50, 284)	161 (45, 410)	44 (0, 174)	182 (25, 477)	70 (7, 204)	69 (0, 201)

[a] LPEP started in 2017 in Brazil and Sri Lanka
Note: Mean of 1,000 runs; 95% prediction interval provided between brackets

Fig N disentangles the impact of contact tracing only and contact tracing with SDR-PEP. Fig O illustrates the associated number of contacts who have received SDR per year for each study area.

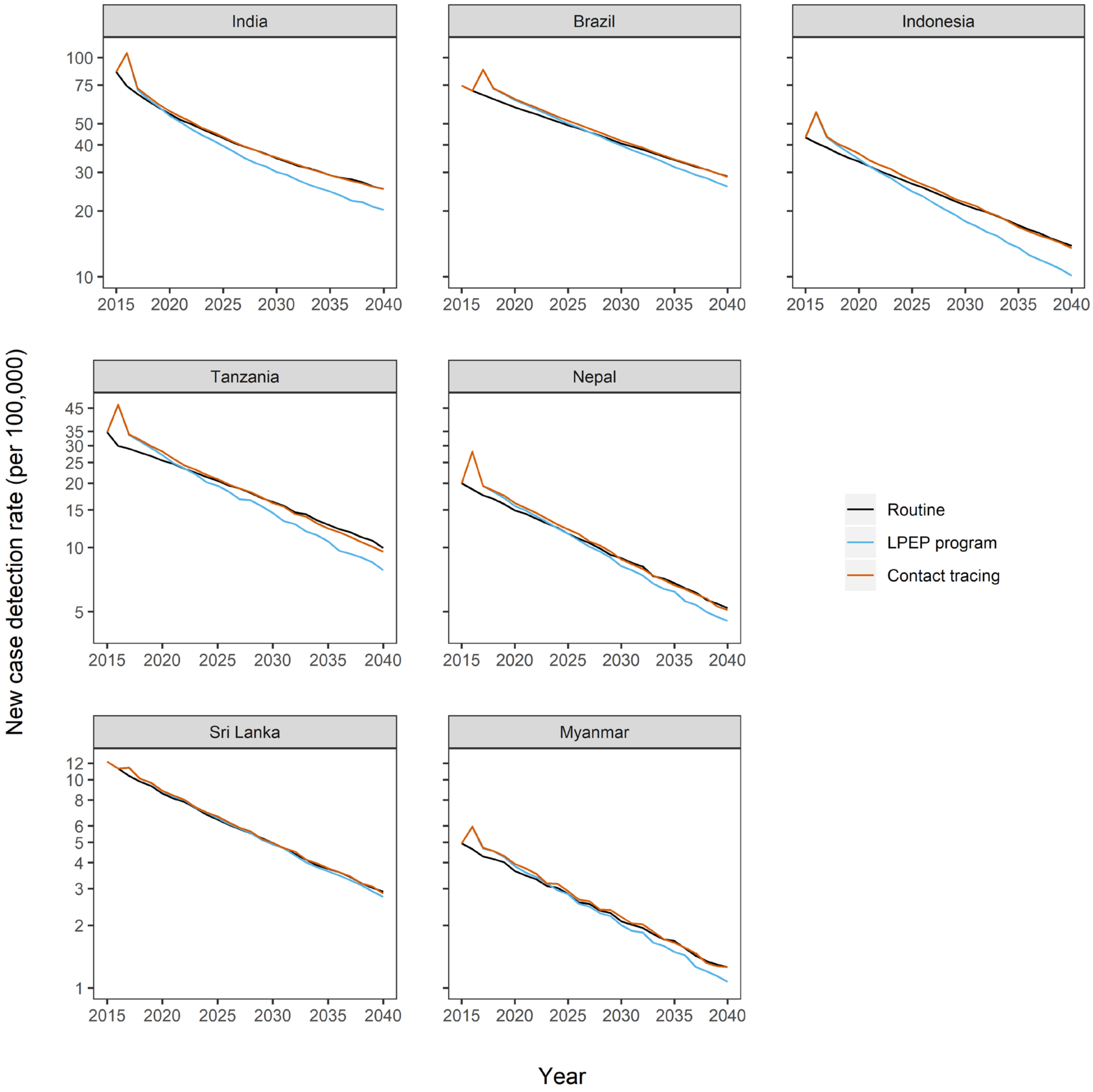

Fig N. Predicted long term trends of the leprosy new case detection rate under the routine programme, and combined with contact tracing only and the LPEP program, stratified by LPEP area. Model predictions are represented by means of 1,000 repeats (solid line). The blue line represents the LPEP program and the black line the routine programme.

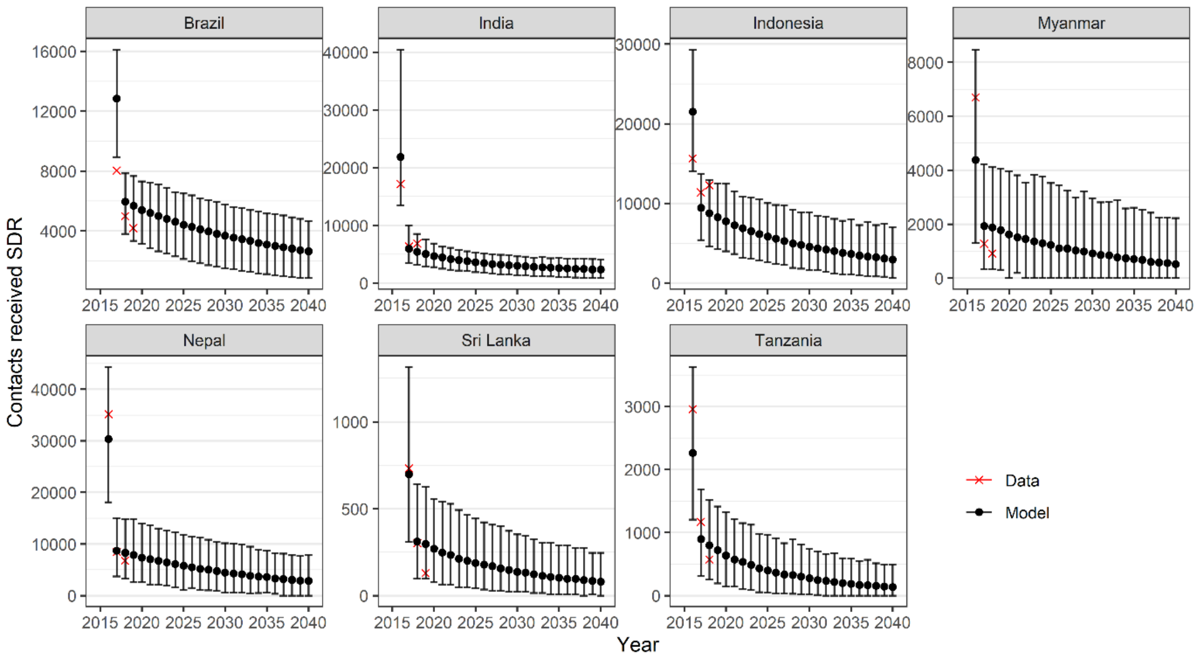

Fig O. Contacts received SDR by year by LPEP study area. Model outcomes are represented by means (points) and 95% prediction intervals (error bars). Data are presented by red crosses. Predictions were made for the years 2015 to 2040. The cumulative number of contacts receiving SDR of all areas together is 558,000 (95% prediction interval: 227,000-1,001,000).

References

1. Blok DJ, de Vlas SJ, Fischer EA, Richardus JH. Mathematical modelling of leprosy and its control. Adv Parasitol. 2015;87:33-51. Epub 2015/03/15. doi: S0065-308X(14)00003-7 [pii]

10.1016/bs.apar.2014.12.002. PubMed PMID: 25765193.
2. Blok DJ, De Vlas SJ, Richardus JH. Global elimination of leprosy by 2020: Are we on track? Parasit Vectors. 2015;8:548. Epub 2015/10/23. doi: 10.1186/s13071-015-1143-4

10.1186/s13071-015-1143-4 [pii]. PubMed PMID: 26490878; PubMed Central PMCID: PMC4618543.
3. de Matos HJ, Blok DJ, de Vlas SJ, Richardus JH. Leprosy new case detection trends and the future effect of preventive interventions in Para State, Brazil: A modelling study. PLoS Negl Trop Dis. 2016;10(3):e0004507. Epub 2016/03/05. doi: 10.1371/journal.pntd.0004507

PNTD-D-15-01874 [pii]. PubMed PMID: 26938738; PubMed Central PMCID: PMC4777416.
4. Fischer E, De Vlas S, Meima A, Habbema D, Richardus J. Different mechanisms for heterogeneity in leprosy susceptibility can explain disease clustering within households. PLoS One. 2010;5(11):e14061. Epub 2010/12/03. doi: 10.1371/journal.pone.0014061. PubMed PMID: 21124916; PubMed Central PMCID: PMC2988824.
5. Fischer EA, de Vlas SJ, Habbema JD, Richardus JH. The long-term effect of current and new interventions on the new case detection of leprosy: a modeling study. PLoS Negl Trop Dis. 2011;5(9):e1330. Epub 2011/09/29. doi: 10.1371/journal.pntd.0001330

PNTD-D-11-00071 [pii]. PubMed PMID: 21949895; PubMed Central PMCID: PMC3176744.
6. Gilkison C, Chambers S, Blok DJ, Richardus JH, Timeon E, Rimon E, et al. Predicting the impact of household contact and mass chemoprophylaxis on future new leprosy cases in South Tarawa, Kiribati: A modelling study. PLoS Negl Trop Dis. 2019;13(9):e0007646. Epub 2019/09/21. doi: 10.1371/journal.pntd.0007646

PNTD-D-19-00347 [pii]. PubMed PMID: 31539374; PubMed Central PMCID: PMC6754131 following competing interests: SC and PP are board members of the Pacific Leprosy Foundation.
7. Blok DJ, de Vlas SJ, Geluk A, Richardus JH. Minimum requirements and optimal testing strategies of a diagnostic test for leprosy as a tool towards zero transmission: A modeling study. PLoS Negl Trop Dis. 2018;12(5):e0006529. doi: 10.1371/journal.pntd.0006529. PubMed PMID: 29799844; PubMed Central PMCID: PMCPMC5991769.
8. Blok DJ, Crump RE, Sundaresh R, Ndeffo-Mbah M, Galvani AP, Porco TC, et al. Forecasting the new case detection rate of leprosy in four states of Brazil: A comparison of modelling approaches. Epidemics. 2017;18:92-100. Epub 2017/03/11. doi: S1755-4365(16)30072-X [pii]

10.1016/j.epidem.2017.01.005. PubMed PMID: 28279460; PubMed Central PMCID: PMC6198811.
9. Blok DJ, de Vlas SJ, Richardus JH. Finding undiagnosed leprosy cases. Lancet Infect Dis. 2016;16(10):1113. Epub 2016/09/28. doi: S1473-3099(16)30370-X [pii]

10.1016/S1473-3099(16)30370-X. PubMed PMID: 27676349.
10. IBGE. Censuses historical data: Instituto Brasileiro de Geografia e Estatística; 2010 [cited 2019 May 23]. Available from: https://ww2.ibge.gov.br/english/estatistica/populacao/censohistorico/default.shtm.
11. IBGE. Censo demográfico 2010: Instituto Brasileiro de Geografia e Estatística; 2010 [cited 2019 May 23]. Available from: http://www.ibge.gov.br/home/estatistica/populacao/censo2010.
12. IBGE. Censo demográfico 2000: Instituto Brasileiro de Geografia e Estatística; 2000 [cited 2019 May 23]. Available from: ftp://ftp.ibge.gov.br/Censos/Censo_Demografico_2000.
13. IBGE. Censo demográfico 1991: Instituto Brasileiro de Geografia e Estatística; 1991 [cited 2019 May 23]. Available from: https://biblioteca.ibge.gov.br/index.php.
14. National Research Council. Levels and recent trends in fertility and mortality in Brazil. Washington, D.C.: Committee on population and demography, 1983.
15. World Health Organization. Life tables by country (Brazil): WHO; [cited 2016 June]. Available from: http://apps.who.int/gho/data/?theme=main&vid=60220.
16. IBGE. Censo demográfico 1980: Instituto Brasileiro de Geografia e Estatística; 1980 [cited 2019 May 23]. Available from: https://biblioteca.ibge.gov.br/index.php.
17. Census India. Variation in population since 1901: Ministry of Home Affairs, Government of India; [cited 2018 July 11]. Available from: http://censusindia.gov.in/Census_Data_2001/India_at_glance/variation.aspx.

18. Census India. Sample Registration System: Ministry of Home Affairs, Government of India; [cited 2018 July 11]. Available from: http://www.censusindia.gov.in/2011-Common/Sample_Registration_System.html.
19. Census India. Tabulations plan of census year - 2011: Ministry of Home Affairs, Government of India; [cited 2018 July 11]. Available from: http://www.censusindia.gov.in/DigitalLibrary/TablesSeries2001.aspx.
20. Nitisastro W. Population trends in Indonesia. US: Cornell University Press; 1970.
21. BPS Statistics Indonesia. Population of Indonesia by province 1971, 1980, 1990, 1995 , 2000 and 2010: Badan Pusat Statistik; [cited 2019 June]. Available from: https://www.bps.go.id/statictable/2009/02/20/1267/penduduk-indonesia-menurut-provinsi-1971-1980-1990-1995-2000-dan-2010.html.
22. World Health Organization. Life tables by country (Indonesia): WHO; [cited 2019 June]. Available from: http://apps.who.int/gho/data/view.main.60750?lang=en.
23. Statistics Indonesia (Badan Pusat Statistik—BPS), National Population and Family Planning Board (BKKBN), Kementerian Kesehatan (Kemenkes—MOH), International I. Indonesia Demographic and Health Survey 2012. Jakarta, Indonesia: BPS, BKKBN, Kemenkes, and ICF International, 2013.
24. UN data. Population by marital status, age, sex and urban/rural residence 2010 [cited 2019 June]. Available from: http://data.un.org/Data.aspx?d=POP&f=tableCode%3A23.
25. United Nations Population Division. World population prospects [cited 2019 March]. Available from: https://population.un.org/wpp/.
26. World Health Organization. Life tables by country (Myanmar): WHO; [cited 2019 March]. Available from: http://apps.who.int/gho/data/view.main.61130?lang=en.
27. Ministry of Labor IaP. The 2014 Myanmar population and housing census. Nay Pyi Taw, Myanmar: Ministry of Labor, Immigration and Population, 2015.
28. Central Bureau of Statistics. Population monograph of Nepal. Kathmandu, Nepal: Government of Nepal National Planning Commission Secretariat, 2014.
29. World Health Organization. Life tables by country (Nepal): WHO; [cited 2019 February]. Available from: http://apps.who.int/gho/data/view.main.LT62120?lang=en.
30. Ministry of Health and Population. Nepal Demographic and Health Survey 2011. Kathmandu, Nepal: Population Division Ministry of Health and Population 2012.
31. Department of Census & Statistics Census of population and housing 2012. Department of Census & Statistics 2015.
32. WHO. Life tables by country (Sri Lanka): WHO; [cited 2019 April]. Available from: http://apps.who.int/gho/data/view.main.LT62220?lang=en.
33. Department of Census and Statistics. Sri Lanka Demographic and Health Survey 2006-07 Colombo, Sri Lanka Ministry of Healthcare and Nutrition 2009.
34. Department of Census and Statistics. Women and men in Sri Lanka. 2012.
35. Agwanda A., Amani H. Population growth, structure and momentum in Tanzania. The Economic and Social Research Foundation (ESRF), 2014.
36. WHO. Life tables by country (Tanzania): WHO; [cited 2018 November]. Available from: http://apps.who.int/gho/data/view.main.61770?lang=en.
37. National Bureau of Statistics Tanzania Demographic and Health Survey 2010. Dar es Salaam, Tanzania National Bureau of Statistics 2011.
38. National Bureau of Statistics. 2012 population and housing census. Ministry of Finance, 2013.
39. National Bureau of Statistics. Basic demographic and socio-economic profile Ministry of Finance, 2014.
40. Meima A, Gupte MD, van Oortmarssen GJ, Habbema JD. SIMLEP: a simulation model for leprosy transmission and control. Int J Lepr Other Mycobact Dis. 1999;67(3):215-36. Epub 1999/11/27. PubMed PMID: 10575401.
41. Programme) NNLE. NLEP - Progress report New Delhi: Central Leprosy Division, 2017.
42. Fine PE. Leprosy: the epidemiology of a slow bacterium. Epidemiological Review. 1982;4:161-88. PubMed PMID: 6754406.
43. Sirumban P, Kumar A, Neelan PN. Healing time in untreated paucibacillary leprosy: a cross-sectional study. Int J Lepr Other Mycobact Dis. 1988;56(2):223-7. Epub 1988/06/01. PubMed PMID: 3261765.
44. Meima A, Smith WC, van Oortmarssen GJ, Richardus JH, Habbema JD. The future incidence of leprosy: a scenario analysis. Bull World Health Organ. 2004;82(5):373-80. PubMed PMID: 15298228; PubMed Central PMCID: PMC2622833.
45. Becxbleumink M. Relapses among Leprosy Patients Treated with Multidrug Therapy - Experience in the Leprosy Control Program of the All Africa Leprosy and Rehabilitation Training-Center (Alert) in Ethiopia - Practical Difficulties with Diagnosing Relapses - Operational Procedures and Criteria for Diagnosing Relapses. Int J Leprosy. 1992;60(3):421-35. PubMed PMID: WOS:A1992KB11300012.

46. Meima A, Smith WCS, Van Oortmarssen GJ, Richardus JH, Habbema JDF. The future incidence of leprosy: a scenario analysis. B World Health Organ. 2004;82(5):373-80. PubMed PMID: WOS:000221566200010.
47. Schuring RP, Richardus JH, Pahan D, Oskam L. Protective effect of the combination BCG vaccination and rifampicin prophylaxis in leprosy prevention. Vaccine. 2009;27(50):7125-8. Epub 2009/09/30. doi: S0264-410X(09)01388-7 [pii]

10.1016/j.vaccine.2009.09.054. PubMed PMID: 19786134.
48. data U. BCG immunization coverage among 1-year-olds (%): United Nations; [cited 2018 November]. Available from: http://data.un.org/Data.aspx?q=IMMUNIZATION&d=WHO&f=MEASURE_CODE%3AWHS4_543.
49. Barth-Jaeggi T, Steinmann P, Mieras L, van Brakel W, Richardus JH, Tiwari A, et al. Leprosy Post-Exposure Prophylaxis (LPEP) programme: study protocol for evaluating the feasibility and impact on case detection rates of contact tracing and single dose rifampicin. BMJ Open. 2016;6(11):e013633. Epub 2016/11/20. doi: bmjopen-2016-013633 [pii]

10.1136/bmjopen-2016-013633. PubMed PMID: 27856484; PubMed Central PMCID: PMC5128948.
50. Moet FJ, Pahan D, Oskam L, Richardus JH, Group CS. Effectiveness of single dose rifampicin in preventing leprosy in close contacts of patients with newly diagnosed leprosy: cluster randomised controlled trial. BMJ. 2008;336(7647):761-4. Epub 2008/03/12. doi: bmj.39500.885752.BE [pii]

10.1136/bmj.39500.885752.BE. PubMed PMID: 18332051; PubMed Central PMCID: PMC2287265.
